# *Burkholderia cepacia* Complex Species Differ in the Frequency of Variation of the Lipopolysaccharide O-Antigen Expression During Cystic Fibrosis Chronic Respiratory Infection

**DOI:** 10.3389/fcimb.2019.00273

**Published:** 2019-07-31

**Authors:** A. Amir Hassan, Carla P. Coutinho, Isabel Sá-Correia

**Affiliations:** ^1^iBB - Institute for Bioengineering, Instituto Superior Técnico, Universidade de Lisboa, Lisbon, Portugal; ^2^Department of Bioengineering, Instituto Superior Técnico, Universidade de Lisboa, Lisbon, Portugal

**Keywords:** *Burkholderia cepacia* complex species, cystic fibrosis, chronic pulmonary infections, lipopolysaccharide O-antigen, within-host clonal variation

## Abstract

*Burkholderia cepacia* complex (Bcc) bacteria can adapt to the lung environment of cystic fibrosis (CF) patients resulting in the emergence of a very difficult to eradicate heterogeneous population leading to chronic infections associated with rapid lung function loss and increased mortality. Among the important phenotypic modifications is the variation of the lipopolysaccharide (LPS) structure at level of the O-antigen (OAg) presence, influencing adherence, colonization and the ability to evade the host defense mechanisms. The present study was performed to understand whether the loss of OAg expression during CF infection can be considered a general phenomenon in different Bcc species favoring its chronicity. In fact, it is still not clear why different Bcc species/strains differ in their ability to persist in the CF lung and pathogenic potential. The systematic two-decade-retrospective-longitudinal-screening conducted covered 357 isolates retrieved from 19 chronically infected patients receiving care at a central hospital in Lisbon. The study involved 21 Bcc strains of six/seven Bcc species/lineages, frequently or rarely isolated from CF patients worldwide. Different strains/clonal variants obtained during infection gave rise to characteristic OAg-banding patterns. The two most prevalent and feared species, *B. cenocepacia* and *B. multivorans*, showed a tendency to lose the OAg along chronic infection. *B*. *cenocepacia recA* lineage IIIA strains known to lead to particularly destructive infections exhibit the most frequent OAg loss, compared with lineage IIIB. The switch frequency increased with the duration of infection and the level of lung function deterioration. For the first time, it is shown that the rarely found *B. cepacia* and *B. contaminans*, whose representation in the cohort of patients examined is abnormally high, keep the OAg even during 10- or 15-year infections. Data from co-infections with different Bcc species reinforced these conclusions. Concerning the two other rarely found species examined, *B*. *stabilis* exhibited a stable OAg expression phenotype over the infection period while for the single clone of the more distantly related *B*. *dolosa* species, the OAg-chain was absent from the beginning of the 5.5-year infection until the patient dead. This work reinforces the relevance attributed to the OAg-expression switch suggesting marked differences in the various Bcc species.

## Introduction

Chronic respiratory infections involving opportunistic pathogens remain the leading cause of premature death of cystic fibrosis (CF) patients (Cullen and McClean, 2015), in particular the long-term infections caused by *Pseudomonas aeruginosa* and bacteria of the *Burkholderia cepacia* complex (Bcc). The Bcc is a group of more than 20 closely-related species (Depoorter et al., 2016) particularly feared by CF patients due to their very difficult eradication, inter-patient transmission, and related increased morbidity and decreased life expectancy (Mahenthiralingam et al., 2005; Drevinek and Mahenthiralingam, 2010; Kenna et al., 2017). The majority of CF patients who acquire Bcc develops a chronic infection while a minority may clear the infection spontaneously (Mahenthiralingam et al., 2005). Pulmonary colonization with Bcc can cause an accelerated decline in lung function and the “cepacia syndrome” that is characterized by an uncontrolled deterioration with septicaemia and necrotizing pneumonia that usually results in early death (Mahenthiralingam et al., 2005). Inside the CF lung, Bcc bacteria face stressful and changing environmental conditions as a consequence of host immune defenses, inflammatory responses, antimicrobials and reactive oxygen species (ROS), fluctuating levels of nutrients, oxygen deprivation, high osmolarity, low pH, biofilm growth, and the presence of other co-infecting microbes (Hogardt and Heesemann, 2010; Döring et al., 2011; Cullen and McClean, 2015). Consequently, during long-term infection, the initial Bcc strain-population accumulates genetic alterations leading to genotypic and phenotypic diversification forming a heterogeneous bacterial community very difficult to eradicate therapeutically (Lieberman et al., 2011, 2014; Madeira et al., 2011, 2013; Silva et al., 2011, 2016; Moreira et al., 2014; Nunvar et al., 2017). Such within-patient emergence of multiple clonal variants was first described for the more prevalent CF pathogen *Pseudomonas aeruginosa* (Markussen et al., 2014; Winstanley et al., 2016) and proposed to provide a pool of mutations affecting virulence and antimicrobial resistance (Lorè et al., 2012; Marvig et al., 2013; Faure et al., 2018). Several studies have shown that, during chronic infection, alterations in the lipopolysaccharide (LPS) molecule, a complex glycolipid covering the cell surface and shielding Gram-negative bacteria from adverse host environments, may occur (Pier, 2007; Maldonado et al., 2016; Faure et al., 2018). The LPS is one of the most important components of pathogenic Gram-negative bacteria cellular envelope being considered a major virulence factor and contributing to adhesion, immune defenses evasion, host colonization and adaptation to the infection niche (Vinion-Dubiel and Goldberg, 2003; Pier, 2007; Maldonado et al., 2016). The LPS structure is composed by three constituents: a highly acylated lipid A, also known as endotoxin, covalently linked to the central core oligosaccharide, and the O-antigen (OAg), composed by repeating polysaccharide units of variable length (Vinion-Dubiel and Goldberg, 2003). Most of the heterogeneity in LPS molecules is found in the OAg polysaccharide and lipid-A components (Pier, 2007; Maldonado et al., 2016; Faure et al., 2018). The OAg component of the LPS is essential in several pathogens for motility (swarming and twitching) (Toguchi et al., 2000; Berry et al., 2009; Bowden et al., 2013), protection against oxidative stress (Berry et al., 2009) and evasion from host immune defenses (Murray et al., 2003; Saldias et al., 2009; Kotrange et al., 2011; Kintz et al., 2017). LPS OAg is believed to be an immunodominant molecule that can modulate host-pathogen interaction (Ranf, 2016) and proposed to be under selective pressure in Gram-negative bacteria (King et al., 2009; Maldonado et al., 2016; Kintz et al., 2017). Longitudinal studies of *P. aeruginosa* isolates from CF patients with chronic lung infections revealed the conversion from smooth (in the early isolates) to rough LPS with short or no OAg side chain at the late-stage of infections rendering the bacteria non-typable and less immunogenic (King et al., 2009; Maldonado et al., 2016; Demirdjian et al., 2017). In Bcc bacteria, a few recent reports also described the variation of the presence and/or modification of the OAg during persistent infection of the CF lung and on how this alteration may affect Bcc pathogenicity (Lieberman et al., 2011; Maldonado et al., 2016; Silva et al., 2016; Hassan et al., 2017). An extensive study on an epidemic outbreak of *Burkholderia dolosa* during 16 years, involving 14 CF patients and 112 isolates, has shown that several late isolates produce an LPS exhibiting the OAg that was absent in the LPS of the ancestral strain; this fact was related with the appearance of two different mutations in the glycosyltransferase encoding gene *wbaD* (Lieberman et al., 2011). The genome analysis of clones and metagenomes of evolved *B. cenocepacia* biofilms, after more than one thousand generations, unveiled a mutation in the gene *manC* – a gene involved in mannose metabolism located within the LPS biosynthesis gene cluster-resulting in the loss of the LPS OAg (Traverse et al., 2013). Remarkably, mutations in the same *manC* gene were previously found to disrupt OAg biosynthesis in late *B. cenocepacia* (Saldias et al., 2009) or early *B. dolosa* (Lieberman et al., 2011) isolates retrieved during chronic infection. A comparative genomic analysis focused on sequential *B. multivorans* isolates obtained over 20 years of CF chronic infection showed that late isolates accumulate three different mutations in a locus homologous to the *wbi* gene cluster, involved in LPS OAg biosynthesis, leading to OAg loss (Silva et al., 2016). Another recent study involving eleven serial clonal variants of *B. cenocepacia recA* lineage IIIA obtained from a CF patient over a 3.5 year period, since the onset of infection until death with the cepacia syndrome, revealed that the OAg was only present in the early isolate but not in any of the late variants (Hassan et al., 2017). Genomic sequencing of these serial isolates enabled the identification of mutations, also within the OAg cluster, but none of these mutations could definitively be associated to OAg loss (Hassan et al., 2017).

Although the Bcc comprises 24 bacterial species (De Smet et al., 2015; Depoorter et al., 2016; Ong et al., 2016; Bach et al., 2017; Weber and King, 2017; Martina et al., 2018) and several were found to be involved in CF respiratory infections, only *B. cenocepacia* (Hassan et al., 2017), *B. multivorans* (Silva et al., 2016), and *B. dolosa* (Lieberman et al., 2011) were examined concerning the variation of the OAg presence over chronic infection. *B. cenocepacia* is recognized as the dominant species with high potential for inter-patient transmission (Drevinek and Mahenthiralingam, 2010), and *B. multivorans* has recently replaced *B. cenocepacia* in this first position in several countries (Lipuma, 2010). However, it is recognized that the less represented species may also be associated with poor clinical outcome, but it is not clear why different Bcc species/strains differ in their ability to persist in the CF lung and in their pathogenic potential (Woods et al., 2004; Kalish et al., 2006; Cunha et al., 2007; Moehring et al., 2014; Coutinho et al., 2015; Nunvar et al., 2016; Marquez et al., 2017; Roux et al., 2017). It also remains to be elucidated whether LPS OAg loss confers an advantage to the different Bcc species in particular and to Gram-negative bacteria, in general, over the course of infection.

The present study was performed to understand whether OAg loss in different Bcc species during respiratory infection in CF patients can be considered a general phenomenon that affects immune evasion favoring chronic infection. For this, a systematic retrospective and longitudinal screening was performed based on a collection of isolates, recovered from 1995 to 2016 from 19 CF patients under surveillance at the major Portuguese CF treatment center at Hospital de Santa Maria (HSM), in Lisbon, over the duration of chronic infection (ranging from 1.2 to 15.2 years). The 357 sequential isolates examined were molecularly identified at the species level and genotyped during this study or in previous studies and belong to *B. cenocepacia, B. multivorans* or to the more rare species in CF population worldwide, *B. dolosa, B. stabilis, B. cepacia* and *B. contaminans* (Cunha et al., 2003, 2007; Coutinho et al., 2011, 2015; Moreira et al., 2014). The abnormally high representation of the species *B. cepacia* and *B. contaminans* in our collection is considered related with a contamination of saline solutions for nasal application (Cunha et al., 2007; Coutinho et al., 2015). Information on the health condition of several patients at the time of isolation based on the forced expiratory values (FEV), an indicator of pulmonary function and, consequently, of oxygen availability in the patient's lung, is also available (Correia et al., 2008; Moreira et al., 2014). This systematic retrospective study allowed to answer the question of whether the described variation of the LPS OAg expression for a limited number of the Bcc species/strains and patients is extensive to the more rarely found Bcc species and can be considered a general phenomenon in the Bcc during long-term respiratory infections in CF patients.

## Materials and Methods

### Bacterial Isolates and Growth Conditions

The 357 *Burkholderia cepacia* complex (Bcc) isolates examined in this study were retrieved from the respiratory secretions of 19 chronically infected cystic fibrosis (CF) patients under surveillance at the major Portuguese CF treatment center at Hospital de Santa Maria (HSM), Centro Hospitalar Lisboa Norte EPE, Lisbon, from 1995 to 2016, during hospital routines. The isolates examined in this retrospective study were selected at random among the colonies isolated in selective *Burkholderia cepacia* Selectatab medium at the Hospital, at a specific date of isolation. Over the years, a significant part of the isolates were molecularly confirmed to belong to six Bcc species: *Burkholderia cenocepacia, Burkholderia multivorans, Burkholderia cepacia, Burkholderia contaminans, Burkholderia dolosa*, and *Burkholderia stabilis* and genotyped (Cunha et al., 2003, 2007; Coutinho et al., 2011, 2015) (Table 1 and Table S1). Bacterial cultures are stored at −80°C in 1:1 (v/v) glycerol. During this study, bacteria were grown in Lysogeny broth agar (LB agar; Conda, Pronadisa) at 37°C with shaking at 250 rpm or in LB agar plates.

**Table 1:** Schematic representation of the sequential isolates from various species chronically infecting the different CF patients examined in this study.

**CF patients**	**Number of sequential isolates with ( 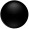 ) or without ( 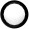 ) O-antigen**	**O-antigen profiles (in Figure 2)**	**Isolate ribo-patterns (R)/RAPD profiles**	**Patient clinical outcome**	**Duration of the chronic infection with the Bcc strain (years)**
***Burkholderia cenocepacia IIIA***					
I	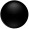 3	A1	R08/RAPD01	Infection eradicated	2.6
J	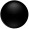 1 * 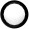 * 10	A2 and A3	R11/RAPD02	Dead ^*^	3.4
O	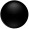 10	A4	R11/RAPD03	Infection eradicated before death	3
P	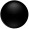 3	A5	R13/RAPD04	Infection eradicated before death	2.5
AB	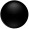 17 * 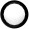 * 29	A6 and A7	R07/RAPD05	Still infected	14.7
AF	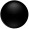 2 * 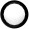 * 5 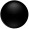 1	A8 and A10 A9	ND/RAPD06 RAPD07	Dead	2.5 0.1
AN	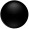 10 * 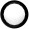 * 21	A11 and A12	R21/RAPD08	Dead	6.8
***Burkholderia cenocepacia IIIB***					
O	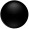 8 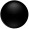 2	B1 B2	R14/RAPD09 RAPD10	Infection eradicated before death	3.4 0.7
R	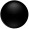 12 * 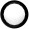 * 2 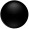 11	B3 and B4	R15/RAPD10	Infection eradicated	7.5
T	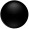 6	B5	R16/RAPD11	Infection eradicated before death	1.2
***Burkholderia cepacia***					
D	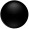 11	C1	ND/RAPD12	Dead	4.4
N	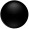 28	C2	R12	Dead	3.8
O	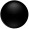 24	C2	R12	Infection eradicated before death	5.6
AL	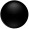 10	C1	R19/RAPD12	Infection eradicated	2.2
AP	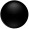 5	C1	R19	Infection eradicated	2.8
AQ	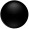 10	C5	R24	Infection eradicated	2.9
AU	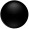 32 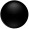 2	C3 C4	ND/RAPD12 RAPD13	Still infected	9.6 0.4
***Burkholderia contaminans***					
V	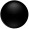 13	D1	R17	Alive	15.2
AF	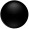 29	D2	R02	Dead	5.8
***Burkholderia multivorans***					
J	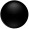 2 * 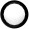 * 6 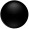 1	E1 and E2	R9	Dead^*^	4
***Burkholderia dolosa***					
AR	* 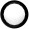 * 14	F	ND	Dead	5.5
***Burkholderia stabilis***					
B	* 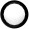 * 4 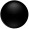 1	G1 G2	R01/RAPD15 RAPD14	Dead	3.8 0.1
H	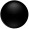 8 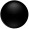 4	G3 G4	R01/RAPD14 RAPD15	Dead^*^	2.5 2

Information on the number of sequential clonal isolates in which the OAg is present (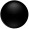) or absent (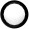) and the corresponding OAg profiles (Figure 2), when present, the ripopatterns of the isolates (Cunha et al., 2003, 2007; Coutinho et al., 2015) and their RAPD profiles (Figure S1) and the duration of the chronic infection and clinical outcome of the infected CF patients (Cunha et al., 2003, 2007; Coutinho et al., 2011; Moreira et al., 2014) is provided. R, Isolate ribopattern; RAPD, Random amplified polymorphism DNA profile (Figure S1); ND, not determined;

* *, cepacia syndrome*.

### Species Identification and Genotyping of Bcc Isolates

The isolates examined in this study that had not been previously identified at the species level and genotyped (by ribotyping or *recA* gene PCR Restriction fragment length polymorphism–*recA* PCR RFLP–and species-specific *recA* direct PCR) were identified and genotyped in this study based on *recA* PCR RFLP analysis and Random Amplified Polymorphic DNA (RAPD) (Mahenthiralingam et al., 1996, 2000). Genomic DNA was extracted from cells of those isolates grown overnight in LB broth with orbital agitation at 37°C, using the Puregene DNA isolation kit (cell and tissue kit, Gentra Systems, Qiagen, Germany). The concentration of the resulting genomic DNA solutions was estimated using a ND-1000 spectrophotometer (NanoDrop Technologies). Species identification was based on the *recA* PCR RFLP analysis that was performed by amplification of the entire *recA* gene using specific primers for Bcc bacteria (BCR1-^5^′TGACCGCCGAGAAGAGCAA^3^′; BCR2-^5^′CTCTTCTTCGTCCATCGCCTC^3^′) (Mahenthiralingam et al., 2000). Bcc *recA* amplicons were digested with *Hae*III (Amersham Biosciences) and the restriction fragments separated by electrophoresis in 2% (w/v) agarose gels. The RFLP patterns obtained were compared with those reported in the literature (Mahenthiralingam et al., 2000; Vermis et al., 2002; Cunha et al., 2003; Coutinho et al., 2015). Strain assignment was performed by RAPD (Mahenthiralingam et al., 1996). Genotyping of the isolates tested was performed using primer 270 (^5^′TGCGCGCGGG^3^′), followed by confirmation of the strain types established by this primer with primers 208 (^5^′ACGGCCGACC^3^′) and 272 (^5^′AGCGGGCCAA^3^′). RAPD profiles were visually compared by GelJ software (Heras et al., 2015). Similarity coefficients were calculated using the RAPD profiles obtained with each primer for the complete set of isolates examined using Pearson coefficient with Arithmetic Mean (UPGMA) linkage method.

### LPS Extraction and SDS-PAGE Analysis

LPS was extracted from cell envelopes obtained from bacterial cells as described before (Marolda et al., 1990) with small modifications (Hassan et al., 2017). Briefly, Bcc cells were harvested from overnight liquid cultures by centrifugation for 1 min after OD_640nm_ adjustment to 2.0 in 1 mL of phosphate buffer saline—PBS (at pH 7.4: 1.8 mM/L KH_2_PO_4_, 137 mM/L NaCl, 2.7 mM/L KCl and 10 mM/L Na_2_HPO_4_). These cells were suspended in 150 μL of lysis buffer, containing 2% SDS, 4% 2-β-mercaptoethanol, and 500 mM Tris-HCl (pH 6.8), and boiled for 10 min. Proteinase K (20 mg/mL) was added, and the samples were incubated at 60°C for 2 h. Finally, samples were mixed with a tracking dye solution (125 mM Tris-HCl [pH 6.8], 2% SDS, 20% [v/v] glycerol, 0.002% bromophenol blue, and 10% mercaptoethanol) and boiled for 5 min before loading the gels. LPS samples were resolved by electrophoresis – at 150 V for about 1 h 40 m – in 14% polyacrylamide gels with a Tricine-SDS system followed by silver staining to visualize the O-antigen (OAg) banding patterns (Marolda et al., 2006).

### Ethics

The present retrospective work involves clinical Bcc isolates obtained as a part of the hospital routine and its use in clinical research was approved by the hospital ethics committee. Consent was obtained from the patient or legal representative for the use of these isolates in research. Patients' anonymity is preserved.

## Results

### During Chronic Infections, *B. cenocepacia recA* Lineage IIIA and *B*. *multivorans* Isolates Were More Prone to Lose the OAg Present in Early Isolates, Compared With *B. cenocepacia recA* Lineage IIIB

**Figure 1:**
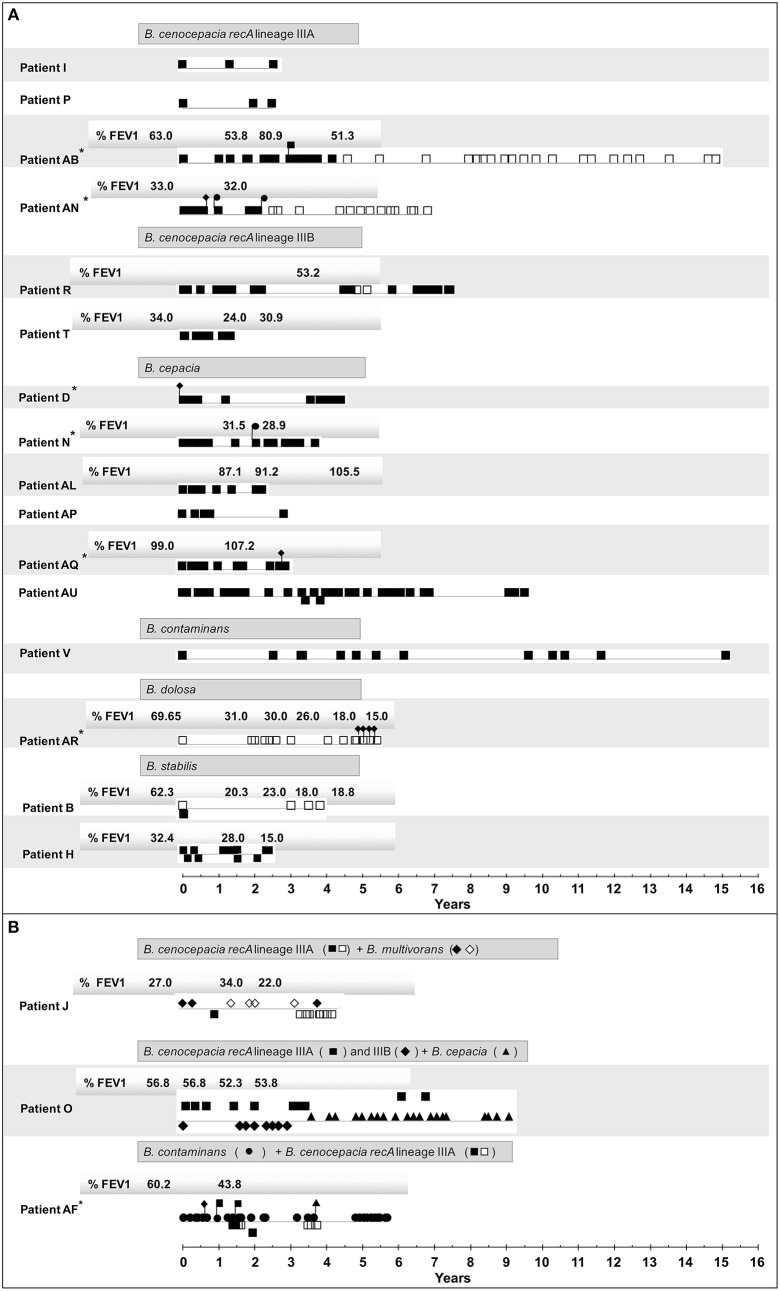
Schematic representation of the Bcc isolates examined in this study retrieved from different CF patients over 21 years (from 1995 to 2016) of epidemiological survey at the CF Center of Hospital de Santa Maria. Time zero marks the date of isolation of the first Bcc isolate from a specific patient with essentially a single Bcc species/strain **(A)** or co-infected with different Bcc species **(B)**. The presence or absence of the LPS O-antigen is represented by closed or open symbols, respectively. The same symbol in a different line is used to indicate a different strain of the same species co-infecting the patient, as detailed in Table 1 and Table S1, as in the case for patients AU, B, H, O, and AF. Whenever available, the Forced Expiratory Values (%) in the first second (FEV1), before and during infection, are provided (Correia et al., 2008; Coutinho et al., 2011; Moreira et al., 2014). These values are an indication of the pulmonary function in the infected patients. ^*^ - Sporadic isolation of strains from other Bcc species (in Table S1): *B. cenocepacia IIIB* -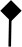, *B. cepacia* -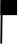, *B. multivorans* -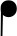, and *B. dolosa* -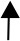.

**Figure 2:**
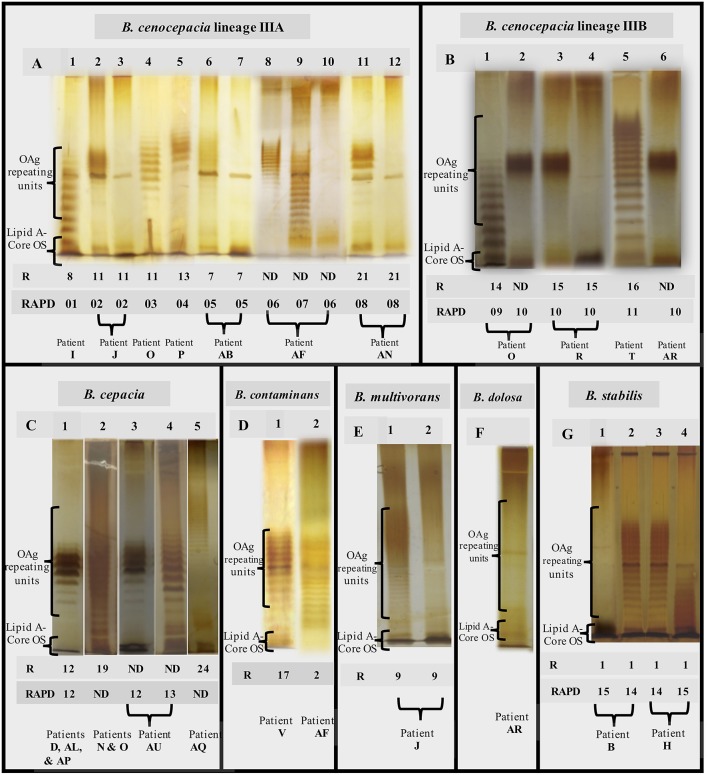
Representative SDS-PAGE gels showing the O-antigen banding patterns obtained (**A1–A12, B1–B6, C1–C5, D1, D2, E1, E2, F, G1–G4**) for the Bcc isolates examined. Genotyping data for the different strains tested are also shown: the ribopatterns were obtained before (Cunha et al., 2003, 2007; Coutinho et al., 2011) and the RAPD profiles were obtained during this study (Figure S1). R, Isolate ribopattern; RAPD, Isolate profile based on Random amplified polymorphism DNA profiles (Figure S1); ND, not determined. The isolates tested are (Table S1): **A1**–IST416, **A2**–IST439, **A3**–IST4103, **A4**–IST462, **A5**–IST432, **A6**–IST4121, **A7**–IST4893, **A8**–IST4240a, **A9**–IST4272, **A10**–IST4253a, **A11**–IST4197, **A12**–IST4386, **B1**–IST435, **B2**–IST4177, **B3**–IST438, **B4**–IST4155, **B5**–IST466, **B6**–IST4481 IIIB, **C1**–IST4152, **C2**–IST4128, **C3**–IST4283, **C4**–IST4546, **C5**–IST4198, **D1**–IST481, **D2**–IST4193, **E1**–IST419, **E2**–IST453, **F**–IST4208, **G1**–IST402, **G2**–IST409, **G3**–IST413, **G4**–IST412.

The 357 *Burkholderia cepacia* complex (Bcc) isolates tested in this study were examined with the goal of systematically compare the O-antigen (OAg) banding patterns when this LPS component is present and to demonstrate the eventual absence of the OAg in late isolates (Figure 1 and Table 1). It was found that the different strains tested and the corresponding clonal variants obtained during long-term-infection, as demonstrated by Multilocus sequence typing (MLST), gave rise to characteristic banding patterns of the OAg whenever this component is present (Figures 2A,B).

Concerning the 153 isolates of *B. cenocepacia recA* lineages IIIA (112 isolates from 7 patients) and IIIB (41 isolates from 3 patients) (Table 1) tested for the presence of OAg polysaccharide during lung infection (timelines shown in Figure 1), it was found that strains from *B. cenocepacia* lineage IIIA were more prone to lose the OAg than lineage IIIB strains.

There were two cases of extended chronic infection of patients AB and AN by *B*. *cenocepacia* lineage IIIA, with a duration of 14.7 and 6.8 years, respectively (Figure 1A and Table 1), where the late isolates retrieved after 4.5 or 2.5 years, respectively, were found to lack the OAg that was present in the early isolates (Figure 1 and Table S1). The loss of the OAg in late isolates was also previously reported for another *B. cenocepacia* lineage IIIA strain infecting patient J for a shorter period of 3.4 years (Hassan et al., 2017) and (Figure 1 and Table 1). Patient J was co-infected with a *B*. *multivorans* strain and the LPS of the majority of late isolates of this *B. multivorans* strain also lack the OAg present in the early isolates, as shown in the electrophoretic profiles of the extracted LPS (Figure 2, columns E1 and E2). However, one *B*. *multivorans* clonal isolate retrieved at the last isolation date exhibited again the OAg, consistent with the pattern observed before by others (Silva et al., 2016). Late isolates of *B*. *cenocepacia* and *B*. *multivorans* were retrieved from patient J during a period of oxygen depletion in the CF lung that accompanied disease progression and the deterioration of lung function. In fact, according to the hospital records, the forced expiratory values in the first second (FEV1) available are low during co-infection (22–34%) and no further values of FEV1 were registered during the later stages of the patient's life due to the highly severe deterioration of pulmonary function (Figure 1B; Correia et al., 2008).

Differently, the *B. cenocepacia* lineage IIIA strain that infected patient O for 3 year-infection period kept the LPS OAg and the same was found for the co-infecting Bcc strains of *B*. *cenocepacia* lineage IIIB and *B. cepacia* chronically infecting patient O for 3.4 and 5.6 years, respectively. Remarkably, the FEV1 values available were above 50% during the co-infection period and, after 9 years, the *Burkholderia* infection was eradicated (Correia et al., 2008; Figure 1, Table S1). When examining the 11 and 10 *B*. *cenocepacia* IIIA isolates obtained from patients' J and O, respectively, we were surprised by the fact that the electrophoretic profiles of the extracted LPS obtained by SDS-PAGE analysis and silver-staining produced two different profiles (Figure 2, columns A2 and A4, respectively) even though they were considered to share the same ribopattern 11 (Table S1; Coutinho et al., 2011). However, the RAPD analysis of the tested isolates revealed that they are clonal isolates but from two different ancestral strains, RAPD02 for patient J and RAPD03 for patient O (Table 1), in line with the different MLST sequence types, ST-218 and ST-280 at the MLST database [http://pubmlst.org/bcc/], obtained for the isolates infecting patient J and O, respectively.

For the *B*. *cenocepacia* IIIA strain co-infecting patient AF for 2.5 years, the loss of the OAg was also observed when the FEV1 values were close to 40%. However, as demonstrated by the different RAPD profiles obtained (Table 1), another *B*. *cenocepacia* IIIA strain was obtained at an isolation date after the loss of the OAg by the original infecting strain; this new strain exhibit an OAg with a distinct banding pattern from the early isolates of the series (Figure 2A, column A9), Remarkably, all the *B*. *contaminans* clonal isolates co-infecting this patient kept the OAg when, for identical isolation times, *B*. *cenocepacia* IIIA clonal isolates lack the OAg (Figure 1B).

Only for *B*. *cenocepacia* IIIA isolates chronically infecting patients I and P over a period of time below 3 years, the loss of OAg along infection was not observed (Figure 1A). Interestingly, after this shorter period of chronic infection, the *Burkholderia* strains were eradicated from both patients.

Concerning the sequential *B. cenocepacia* lineage IIIB isolates retrieved from patient T over 1.2 years of infection before eradication, all the sequential isolates exhibit the OAg even though the FEV1 value was in the low range of 25–30%. In the case of patient R, among the 25 sequential isolates of the *B. cenocepacia* lineage IIIB strain obtained during a very extended infection period (7.5 years) two isolates retrieved after the fifth year of infection were found to lack the OAg (Figures 1, 2, columns B3 and B4, and Table 1). Interestingly, the OAg banding pattern obtained for isolates with ribopattern 15 infecting patient R was identical to the OAg profile extracted from two isolates retrieved from patient O (Figure 2, columns B3 and B2) and identical to those from four *B. cenocepacia recA* lineage IIIB isolates also present in patient AR who was mainly infected with *B. dolosa* (Figure 2, columns B3 and B6). All these isolates producing the same banding pattern were confirmed to share the same RAPD profile (Table 1 and Figure S1). Consistent with these results the MLST database showed that the *B. cenocepacia* lineage IIIB isolates retrieved from patients R and AR also have the same MLST sequence type (ST-43) [http://pubmlst.org/bcc/].

### The OAg Is Stably Expressed in *B. cepacia, B. contaminans* and *B. stabilis*

Regarding the usually less frequent species worldwide that were examined in this study, the *B. cepacia* isolates (a total of 87) retrieved from five patients (N, AL, AP, AQ an AU) infected with this species for a period of time ranging from 2.2 to 9.6 years, all exhibit the OAg even when the FEV1 value was as low as 30% (patient N). Patients AL, AP and AQ, eradicated the infection after the screening period. Interestingly, the OAg banding pattern obtained for isolates with ribopattern 19 that infected patients AL and AP was identical to the OAg profile extracted from isolates retrieved from patients AU and D (Figure 2, columns C1 and C3), the sole exception were the two isolates retrieved from patient AU during the third year of infection with a different RAPD profile (Figures 1, 2, columns C3 and C4, and Table 1). All these isolates producing the same banding pattern were confirmed to share the same RAPD profile (Table 1 and Figure S1).

Concerning the 13 *B. contaminans* clonal isolates retrieved from patient V infected for a very long period of 15.2 years (Cunha et al., 2003; Coutinho et al., 2011), all of them were found to express the OAg (Figure 2, column D1).

Regarding the 17 *B. stabilis* isolates retrieved from patients B and H for a period of 3.8 and 2.5 years of chronic infection, respectively (Figure 1 and Table 1), the two strains isolated from each patient exhibit the same RAPD profiles (Figure S1, RAPD14 and RAPD15, Table 1). Remarkably, a stable phenotype concerning the presence or absence of the OAg over the infection period was found. The four isolates with RAPD15 profile retrieved from patient B exhibit an LPS that lack the OAg, independently of being early or late isolates, while the 4 clonal isolates of the strain with the same RAPD profile obtained from patient H, all have the OAg (Table 1 and Figure 2, columns G1 and G4). Interestingly, patient H died from the cepacia syndrome while patient B death was not related with the cepacia syndrome. Isolates with RAPD14 profile do have an LPS that stably maintained the OAg. When present, the banding patterns of the OAg were different for the two strains but identical for the clonal variants of each strain obtained from both patients (Figure 2, columns G2-G4).

### The LPS From Early and Late *B. dolosa* Isolates Lack the OAg

The analysis of 14 *B. dolosa* isolates obtained from a single patient (AR) chronically infected with this species for 5.5 years showed that this strain did not have an LPS with the OAg chain from the beginning of the infection until dead (Figure 2F). The very peculiar *B*. *dolosa* LPS chemical structure of the first isolate IST4208 obtained from patient AR (Table S1) was examined before and a novel complete structure of the lipooligosaccharide (LOS) lacking the OAg component was revealed (Lorenzo et al., 2013). This LOS was found to show a strong proinflammatory activity (Lorenzo et al., 2013).

## Discussion

Different virulence factors have been described in *Burkholderia cepacia* complex (Bcc) bacteria (Loutet and Valvano, 2010; Sousa et al., 2017) but less is known concerning their adaptive traits during chronic infection, compared with *P. aeruginosa*, the most prevalent and studied CF pathogen (Valentini et al., 2018). Before the systematic retrospective-longitudinal-screening, carried out in the present study, a retrospective phenotypic longitudinal assessment of the variation of mucoid exopolysaccharide production (Zlosnik et al., 2008), the switch from mucoid to non-mucoid (Zlosnik et al., 2011) and the swimming motility (Zlosnik et al., 2014) was performed during chronic infections involving numerous Bcc isolates representing different Bcc species and collected from 100 CF patients in the Vancouver area. Conversion of the mucoid-to-non-mucoid phenotype during chronic infection strengthen the concept of the association of the non-mucoid phenotype with the severity of the disease and the mucoid phenotype with persistence (Zlosnik et al., 2008, 2011). This behavior is in contrast with the phenotypic switch from non-mucoid to mucoid in *P. aeruginosa* chronic infections as a well-established paradigm for infection disease severity and persistence in this species (Valentini et al., 2018). Moreover, upon establishment of chronic infection, subsequent *P. aeruginosa* isolates show a reduction in the swimming ability to be non-motile (Valentini et al., 2018), contrasting with the observed data in Bcc infections (Zlosnik et al., 2014). The understanding of the occurrence of these and other adaptive traits that have been well-described in *P. aeruginosa* chronic infections (Valentini et al., 2018), but are far less known in Bcc chronic infection, is instrumental for a better understanding of bacterial transformations in the CF lungs during long term infections and infection control.

The LPS forms the outer leaflet of the outer membrane and has roles in antigenicity, the inflammatory response and exclusion of external molecules (King et al., 2009). Although the lipid A can vary, the variability in the O-antigen (OAg) is greater influencing adherence, colonization and the ability to evade the host's defense mechanisms (Kintz and Goldberg, 2008; King et al., 2009; Saldias et al., 2009; Kotrange et al., 2011; Maldonado et al., 2016; Ranf, 2016; Kintz et al., 2017). The ability of Bcc species to evade the host immune response often leads to chronic infections that are associated with significant loss of lung function due to the hyperactive inflammatory response that ultimately can lead to fatalities. The systematic, comprehensive and retrospective longitudinal screening conducted in this work, covering multiple sequential isolates from different Bcc strains of species, frequently or rarely isolated from CF patients, to search for the eventual lack of OAg expression in the late-stage of infections, reinforce the relevance that has been attributed to this switch. This was particularly frequent and clear for *B. cenocepacia recA* lineage IIIA and *B. multivorans*, also occurring at lower frequency in *B*. *cenocepacia recA* lineage IIIB. However, in the case of *B. multivorans*, there is a late isolate obtained within the series of sequential cloned isolates lacking the OAg that was found to express it. This behavior was previously observed for *B*. *multivorans* by others (Silva et al., 2016).

For the first time, it was shown that *B*. *cepacia* and *B*. *contaminans* do not lose the OAg even during long term infections that last for periods of time as extended as 9.6 and 15.2 years, respectively. Among the less frequent species, *B. contaminans* is currently considered an emerging CF pathogen (Vanlaere et al., 2009; Martina et al., 2013; Coutinho et al., 2015; Medina-Pascual et al., 2015; Nunvar et al., 2016; Power et al., 2016). Concerning the other two rarely found species examined, *B*. *stabilis* exhibits in two infected patients a stable phenotype concerning the presence or absence of the OAg over the infection period, consistent with the description of *B. stabilis* sp. Nov, as having a relatively stable genome (Vandamme et al., 2000). For the single patient chronically infected for 5.5 years with the more distantly related Bcc species *B. dolosa*, the OAg chain was absent from the beginning of the infection until the patient death. The novel LPS structure of this *B*. *dolosa* strain was examined and its strong pro-inflammatory activity reported before (Lorenzo et al., 2013).

The conclusions from this work were in general supported by the results gathered and suggest that the observed switch frequency of the OAg expression appears to be species dependent and also dependent on the duration of the infection and the level of deterioration of the lung function, increasing with both. This observation is consistent with the fact that cystic fibrosis is a genetic disorder associated with inflammation, sub-optimal antioxidant protection and the continuous use of antimicrobial therapy, all resulting in marked oxidative stress (Galli et al., 2012) that causes an increase in *mutation* rate. Results obtained from co-infected patients with different Bcc species reinforce those conclusions as it is for example the case for patient AF who was infected with *B*. *contaminans* isolates that kept the OAg during 5.8 years of chronic infection while late clonal isolates of the co-infecting *B*. *cenocepacia* IIIA strain during a much shorter period of 2.5 loss the OAg.

It is interesting to note that the two Bcc species that showed a higher tendency to lose the OAg expression along chronic lung infections in CF (*B*. *cenocepacia* and *B*. *multivorans*) are also the two most prevalent worldwide and the most feared among the CF community (Jones et al., 2004; Mahenthiralingam et al., 2005). Remarkably, *B. cenocepacia* and *B. multivorans* cell lysates are more potent stimulators of pro-inflammatory cytokines compared with those from other Bcc species (De Soyza et al., 2004). However, even though the prevalence of *B. multivorans* has recently replaced *B. cenocepacia* in several countries (Baldwin et al., 2008; Lipuma, 2010; Medina-Pascual et al., 2012; Peeters et al., 2017), it is clear that *B. cenocepacia recA* lineage IIIA remains the most dominant species (Manno et al., 2004; Nunvar et al., 2017; Scoffone et al., 2017; Teri et al., 2018). Remarkably, the particularly destructive nature of *B. cenocepacia recA* group IIIA infections in CF patients when compared with other Bcc bacteria has been reported (Manno et al., 2004; Zlosnik et al., 2015).

Remarkably, the single clone of the more distantly related *B*. *dolosa* Bcc species tested did not exhibit the OAg chain from the beginning to the end of the 5.5 year-infection as reported before (Lorenzo et al., 2013). Comprehensive comparative genomic studies performed during chronic infection on the most feared *B*. *cenocepacia* (Nunvar et al., 2017) and *B*. *multivorans* (Silva et al., 2016) and on the far less distributed *B*. *dolosa* (Lieberman et al., 2011); revealed that the genes affected during adaptive evolution in *B*. *cenocepacia* or in *B. multivorans* are different from *B*. *dolosa*. This indicates that evolution in *B*. *dolosa* during chronic CF infection is driven by different selective forces presumably linked to host immune responses (Lieberman et al., 2014; Nunvar et al., 2017). Specifically, while the most mutated genes reported to undergo adaptive within-patient evolution in *B*. *cenocepacia* were associated with oxidative stress response and transition metal metabolism, the same was not observed either in *B*. *dolosa* or in *Pseudomonas aeruginosa* (Lieberman et al., 2014; Nunvar et al., 2017). These genes encode proteins required for protection against the reactive oxygen species (ROS) produced by leukocytes thus suggesting the involvement of the host immune system in driving *B*. *cenocepacia* evolution during chronic CF infection (Nunvar et al., 2017). Given that persistent inflammation and neutrophil infiltration often accompany chronic lung infections, the authors hypothesized that under increased stress encountered in CF macrophages, the global stress response might be activated by the evolved bacterial population and thus modulate the course of infection (Chua et al., 2016, 2017; Nunvar et al., 2017). Since the interactions between pathogens and the immune system are highly complex, affecting pathogen adaptation to different host immune system stimuli, further investigation into the roles of macrophages and defense mechanisms in chronic infection outcome is needed (Chua et al., 2016, 2017).

The presence/absence of the OAg at the Bcc bacteria surface is important for cell evasion from the host immune response and pathogenicity. The OAg is considered highly immunogenic and induces the production of antibodies that may activate the complement pathway, either through the classic pathway or an alternate pathway, which leads to cellular death or phagocytosis (Reyes et al., 2012). Certain modifications in the oligosaccharide chain of the OAg may alter the interaction of the complement pathway. Several OAg of pathogens are similar to host molecules and this facilitate invasion through mimicking in the host (Reyes et al., 2012). An OAg deficient *B*. *cenocepacia* strain was found to be more susceptible to phagocytic internalization (Saldias et al., 2009) while, the loss of OAg expression in *P. aeruginosa* during chronic pulmonary infection did not confer phagocytic resistance *in vitro* (Demirdjian et al., 2017). Given that *Burkholderia* is a non-obligate intracellular pathogen while *P. aeruginosa* is considered an extracellular pathogen, it was hypothesized that the loss of OAg may benefit the ability of *Burkholderia* to access an intracellular environment, having no similar benefits for *P. aeruginosa* (Saldias et al., 2009). Recent studies suggest that there is a high selective pressure on the OAg biosynthetic locus leading to alterations both at the structural, genetic and regulatory levels strongly suggestive of an adaptive mechanism that potentially contribute to evade the host immune defenses in several Gram-negative pathogens (Murray et al., 2003; Yang et al., 2011; Maldonado et al., 2016; McCarthy et al., 2017; Faure et al., 2018; Neiger et al., 2019). Moreover, the OAg absence was shown to increase Bcc survival in eukaryotic cells as amoebae, epithelial cells and human macrophages (Saldias and Valvano, 2009; Saldias et al., 2009; Kotrange et al., 2011; Maldonado et al., 2016), leading to increased internalization of *B*. *cenocepacia* into macrophages upon phagocytosis (Saldias et al., 2009; Kotrange et al., 2011) and to facilitate *B*. *multivorans* growth inside macrophage (Schmerk and Valvano, 2013). These results may suggest that OAg loss could promote Bcc persistence through intracellular survival. Remarkably, the comparison of the ability to subvert the host's immune function, assessed by internalization assays using human dendritic cells, of three of the *B. cenocepacia* isolates examined in this work and retrieved from CF patient J showed that the late variants, IST4113 and IST4134, were significantly more internalized exhibiting increased survival within dendritic cells than the early isolate IST439 (Cabral et al., 2017). This early isolate was the only one that expresses the OAg unit (Hassan et al., 2017) supporting the idea that the loss of the OAg is advantageous for Bcc persistence. In addition, the absence of the OAg in these same isolates was suggested to reduce *B. cenocepacia* virulence potential using the *Galleria mellonella* infection model (Moreira et al., 2017). Such decrease in the virulence potential of early *B. cenocepacia* clonal variants exhibiting the OAg compared with late clonal variants lacking the OAg was also observed for two other chronically infected patients (AB and AN) with *B. cenocepacia* (Moreira et al., 2017). Also, a *Salmonella typhimurium* mutant missing the entire OAg was found to be avirulent in *G. mellonella* infection model while the shortening of the OAg chain length reduced the pathogenic potential by one-half compared to wild-type strain (Bender et al., 2013) suggesting that the OAg length is also a key-determinants of virulence in *G. mellonella*. The OAg truncation has also been implicated in increased neutrophil-mediated killing, complement-mediated susceptibility and phagocytosis of *Salmonella* by macrophages (Murray et al., 2003).

It is essential to obtain comprehensive knowledge about the pathogenesis of the various Bcc species involved in acute or chronic infections in different patients in order to fight the associated infections. This work reinforces the relevance that has been attributed to the switch involved in the lack of expression of the OAg during chronic infection in the most feared pathogenic Bcc species *B. cenocepacia* (in particular the *recA* lineage IIIA) and *B. multivorans*. It also suggests that the evolution of different Bcc species in chronic CF infection also at the level of OAg expression may be driven by different selective forces presumably associated to host immune responses.

## Data Availability

All datasets generated for this study are included in the manuscript/Supplementary Files.

## Author Contributions

AH performed most of the experimental work and CC contributed to the species/lineage identification and genotyping of part of the isolates. IS-C designed and supervised the work and with AH analyzed the data and wrote the manuscript. All the authors approved it.

### Conflict of Interest Statement

The authors declare that the research was conducted in the absence of any commercial or financial relationships that could be construed as a potential conflict of interest.

## Supplementary Material

Figure S1 Representative profiles based on Random Amplified Polymorphic DNA (RAPD) analysis of the Bcc isolates examined in this study retrieved from different CF patients during several years of chronic infection. The polymorphisms shown are based on the amplification by RAPD primers 270 **(A)**, 208 **(B)**, and 272 **(C)** in the same sequential order. The polymorphisms originated were analyzed using the software GelJ to detect the banding patterns. These profiles correspond to the 15-different representative selected isolates of *B. cenocepacia* IIIA (RAPD 01-08), *B. cenocepacia* IIIB (RAPD 09-11), *B. cepacia* (RAPD 12 and 13), and *B. stabilis* (RAPD 14 and 15). RAPD profiles for isolates: **01**–IST416, **02**–IST439, **03**–IST462, **04**–IST432, **05**–IST4121, **06**–IST4240a, **07**–IST4272, **08**–IST4197, **09**–IST435, **10**–IST438, **11**–IST466, **12**–IST4152, **13**–IST4546, **14**–IST413, **15**–IST412 and **λ**−1 Kb plus DNA ladder.

Table S1 Detailed description of the *Burkholderia* sequential isolates per patient examined in this study with their isolation dates. The presence or absence of the LPS OAg is presented by + or – symbols, respectively, and their corresponding profiles (Figure 2) is provided. Information on the species identification and genotyping for the different strains tested are also presented: the genotyping data and the ribopatterns were obtained before (Cunha et al., 2003, 2007; Coutinho et al., 2011, 2015; Moreira et al., 2014, 2017) and the RAPD profiles were obtained in this study (Figure S1) together with the sequence type, when present in the MLST database. The different letter (A, B, C and D) of the isolate signature (as ISTnumber A, B, C or D) was used to indicate different clone at the same isolation date. ND, Not determined.

## Abbreviations

Bcc: *Burkholderia cepacia* complex
CF: cystic fibrosis
HSM, Hospital de Santa Maria: Centro Hospitalar Lisboa Norte
LPS: lipopolysaccharide
OAg: O-antigen
RAPD: random amplified polymorphism DNA
MLST: multilocus sequence typing
ST: MLST sequence type.
