# Supplementary figures and images for: Burkholderia cepacia Complex Species Differ in the Frequency of Variation of the Lipopolysaccharide O-Antigen Expression During Cystic Fibrosis Chronic Respiratory Infection
Source: Front Cell Infect Microbiol. 2019 Jul 31;9:273. doi: 10.3389/fcimb.2019.00273 (PMC6686744; doi:10.3389/fcimb.2019.00273)

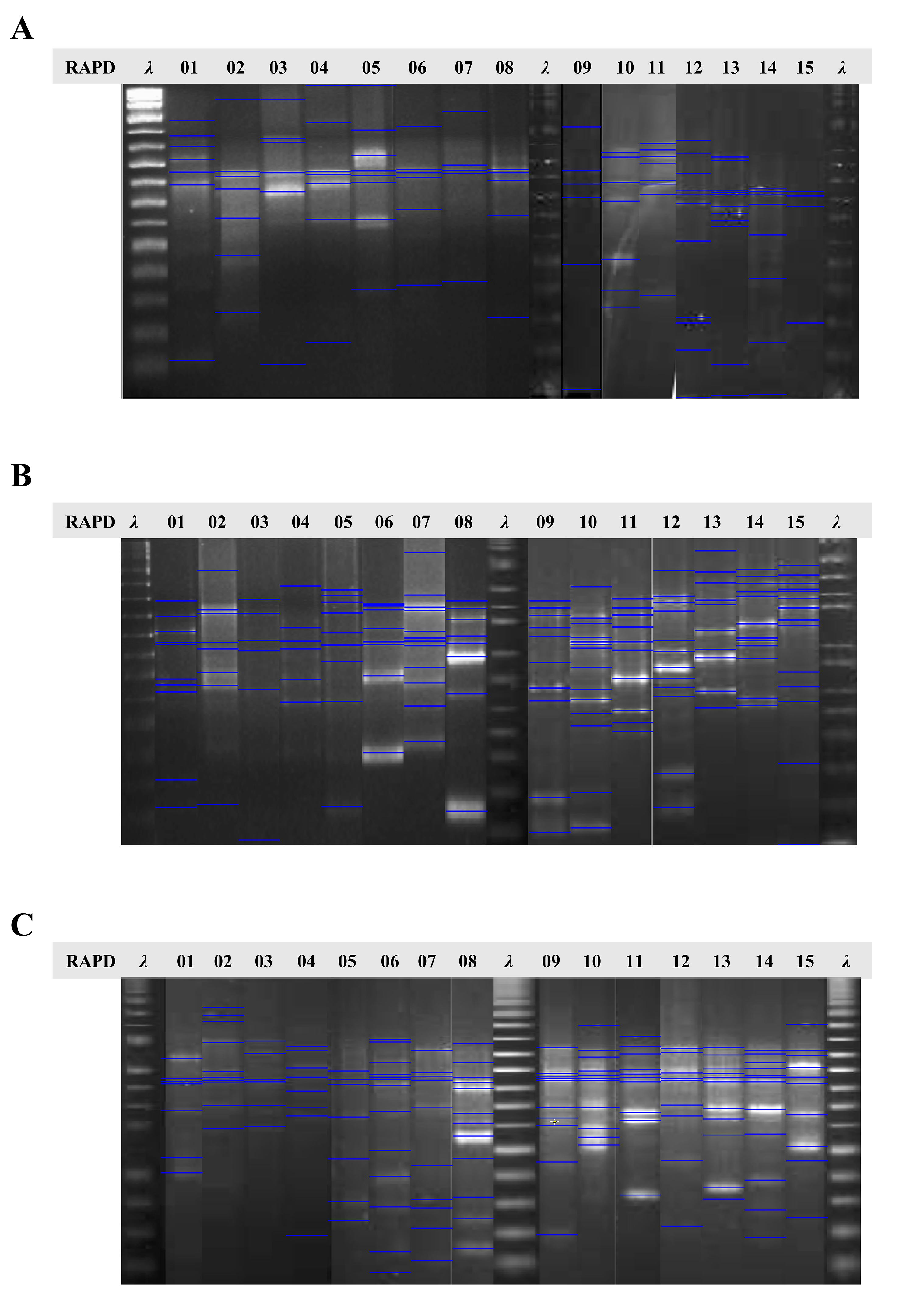

Supplement: Figure S1 — Representative profiles based on Random Amplified Polymorphic DNA (RAPD) analysis of the Bcc isolates examined in this study retrieved from different CF patients during several years of chronic infection. The polymorphisms shown are based on the amplification by RAPD primers 270 (A), 208 (B), and 272 (C) in the same sequential order. The polymorphisms originated were analyzed using the software GelJ to detect the banding patterns. These profiles correspond to the 15-different representative selected isolates of B. cenocepacia IIIA (RAPD 01-08), B. cenocepacia IIIB (RAPD 09-11), B. cepacia (RAPD 12 and 13), and B. stabilis (RAPD 14 and 15). RAPD profiles for isolates: 01–IST416, 02–IST439, 03–IST462, 04–IST432, 05–IST4121, 06–IST4240a, 07–IST4272, 08–IST4197, 09–IST435, 10–IST438, 11–IST466, 12–IST4152, 13–IST4546, 14–IST413, 15–IST412 and λ−1 Kb plus DNA ladder. [file Image_1.JPEG]
