# Supplementary material for: Burkholderia cepacia Complex Species Differ in the Frequency of Variation of the Lipopolysaccharide O-Antigen Expression During Cystic Fibrosis Chronic Respiratory Infection
Source: Front Cell Infect Microbiol. 2019 Jul 31;9:273. doi: 10.3389/fcimb.2019.00273 (PMC6686744; doi:10.3389/fcimb.2019.00273)
Supplement: Table S1 — Detailed description of the Burkholderia sequential isolates per patient examined in this study with their isolation dates. The presence or absence of the LPS OAg is presented by + or – symbols, respectively, and their corresponding profiles (Figure 2) is provided. Information on the species identification and genotyping for the different strains tested are also presented: the genotyping data and the ribopatterns were obtained before (Cunha et al., 2003, 2007; Coutinho et al., 2011, 2015; Moreira et al., 2014, 2017) and the RAPD profiles were obtained in this study (Figure S1) together with the sequence type, when present in the MLST database. The different letter (A, B, C and D) of the isolate signature (as ISTnumber A, B, C or D) was used to indicate different clone at the same isolation date. ND, Not determined. [file Data_Sheet_1.PDF]

# *Supplementary Material*

# *Burkholderia cepacia* complex species differ in the frequency of variation of the lipopolysaccharide O-antigen expression during cystic fibrosis chronic respiratory infection

**A. Amir Hassan, Carla P. Coutinho, Isabel Sá-Correia***

iBB - Institute for Bioengineering and Biosciences, Department of Bioengineering, Instituto Superior Técnico, Universidade de Lisboa, Av. Rovisco Pais, 1049-001 Lisbon, Portugal

*** Correspondence:** Professor Isabel Sá-Correia; isacorreia@tecnico.ulisboa.pt

## Supplementary Tables

**Table S1:** Detailed description of the *Burkholderia* sequential isolates per patient examined in this study with their isolation dates. The presence or absence of the LPS OAg is presented by + or – symbols, respectively, and their corresponding profiles (Figure 2) is provided. Information on the species identification and genotyping for the different strains tested are also presented: the genotyping data and the ribopatterns were obtained before (Cunha et al., 2003; Cunha et al., 2007; Coutinho et al., 2011; Moreira et al., 2014; Coutinho et al., 2015; Moreira et al., 2017) and the RAPD profiles were obtained in this study (Figure S1) together with the sequence type, when present in the MLST database. The different letter (A, B, C and D) of the isolate signature (as ISTnumberA, B, C or D) was used to indicate different clone at the same isolation date.

ND – Not determined.

| Patient | Isolate | Isolation date | species | O-antigen presence | OAg profiles (Fig. 2) | RAPD profiles (Fig.S1) | Isolate ripo-patterns | Sequence type | reference |
|---|---|---|---|---|---|---|---|---|---|
| **Patient B** | IST402 | 4/13/95 | *B. stabilis* | - | G1 | RAPD15 | 1 | ND | Cunha et al., 2003 |
| | IST409 | 4/24/95 | *B. stabilis* | + | G2 | RAPD14 | 1 | ND | Cunha et al., 2003 |
| | IST421 | 4/3/98 | *B. stabilis* | - | G1 | RAPD15 | 1 | ND | Cunha et al., 2003 |
| | IST428 | 9/28/98 | *B. stabilis* | - | G1 | RAPD15 | 1 | ND | Cunha et al., 2003 |
| | IST437 | 1/25/99 | *B. stabilis* | - | G1 | RAPD15 | 1 | ND | Cunha et al., 2003 |
| **Patient D** | IST407 | 12/6/95 | *B. cenocepacia* IIIB | + | - | - | 3 | ND | Cunha et al., 2003 |
| | IST4288A | 1/11/07 | *B. cepacia* | + | C1 | RAPD12 | ND | ND | This work |
| | IST4326A | 4/27/07 | *B. cepacia* | + | C1 | RAPD12 | ND | ND | This work |
| | IST4368A | 6/20/07 | *B. cepacia* | + | C1 | RAPD12 | ND | ND | This work |
| | IST4368B | 6/20/07 | *B. cepacia* | + | C1 | RAPD12 | ND | ND | This work |
| | IST4390A | 3/12/08 | *B. cepacia* | + | C1 | RAPD12 | ND | ND | This work |
| | IST4558A | 7/13/10 | *B. cepacia* | + | C1 | RAPD12 | ND | ND | This work |
| | IST4563A | 10/26/10 | *B. cepacia* | + | C1 | RAPD12 | ND | ND | This work |
| | IST4616A | 2/8/11 | *B. cepacia* | + | C1 | RAPD12 | ND | ND | This work |
| | IST4629A | 3/29/11 | *B. cepacia* | + | C1 | RAPD12 | ND | ND | This work |
| | IST4645A | 5/9/11 | *B. cepacia* | + | C1 | RAPD12 | ND | ND | This work |
| | IST4649A | 6/1/11 | *B. cepacia* | + | C1 | RAPD12 | ND | ND | This work |
| **Patient H** | IST412 | 1/21/97 | *B. stabilis* | + | G3 | RAPD14 | 1 | ND | Cunha et al., 2003 |
| | IST413 | 3/18/97 | *B. stabilis* | + | G4 | RAPD15 | 1 | ND | Cunha et al., 2003 |
| | IST414 | 5/13/97 | *B. stabilis* | + | G3 | RAPD14 | 1 | ND | Cunha et al., 2003 |
| | IST415 | 7/8/97 | *B. stabilis* | + | G4 | RAPD15 | 1 | ND | Cunha et al., 2003 |
| | IST420 | 3/11/98 | *B. stabilis* | + | G3 | RAPD14 | 1 | ND | Cunha et al., 2003 |
| | IST423 | 5/6/98 | *B. stabilis* | + | G3 | RAPD14 | 1 | ND | Cunha et al., 2003 |
| | IST425 | 7/7/98 | *B. stabilis* | + | G3 | RAPD14 | 1 | ND | Cunha et al., 2003 |
| | IST427A | 8/13/98 | *B. stabilis* | + | G3 | RAPD14 | 1 | ND | Cunha et al., 2003 |
| | IST427B | 8/13/98 | *B. stabilis* | + | G4 | RAPD15 | 1 | ND | Cunha et al., 2003 |
| | IST446 | 3/13/99 | *B. stabilis* | + | G4 | RAPD15 | 1 | ND | Cunha et al., 2003 |
| | IST448 | 6/4/99 | *B. stabilis* | + | G3 | RAPD14 | 1 | ND | Cunha et al., 2003 |
| | IST451 | 7/8/99 | *B. stabilis* | + | G3 | RAPD14 | 1 | ND | Cunha et al., 2003 |
| **Patient I** | IST416 | 9/30/97 | *B. cenocepacia* IIIA | + | A1 | RAPD01 | 8 | ND | Cunha et al., 2003 |
| | IST442 | 2/10/99 | *B. cenocepacia* IIIA | + | A1 | RAPD01 | ND | ND | This work |
| | IST464 | 5/24/00 | *B. cenocepacia* IIIA | + | A1 | RAPD01 | ND | ND | This work |
| Patient J | IST419 | 2/26/98 | *B. multivorans* | + | E1 | ND | 9 | ND | Cunha et al., 2003 |
| | IST424 | 6/4/98 | *B. multivorans* | + | E1 | ND | 9 | ND | Cunha et al., 2003 |
| | IST439 | 1/30/99 | *B. cenocepacia* IIIA | + | A2 | RAPD02 | 11 | 218 | Cunha et al., 2003, Coutinho et al., 2011b |
| | IST453 | 7/19/99 | *B. multivorans* | - | E2 | ND | 9 | 375 | Cunha et al., 2003 |
| | IST455A | 2/1/00 | *B. multivorans* | - | E2 | ND | ND | ND | This work |
| | IST455B | 2/1/00 | *B. multivorans* | - | E2 | ND | ND | ND | This work |
| | IST461 | 4/4/00 | *B. multivorans* | - | E2 | ND | 9 | ND | Cunha et al., 2003 |
| | IST495A | 5/29/01 | *B. multivorans* | - | E2 | ND | ND | ND | This work |
| | IST595B | 5/29/01 | *B. multivorans* | - | E2 | ND | ND | ND | This work |
| | IST4103 | 7/24/01 | *B. cenocepacia* IIIA | - | A3 | RAPD02 | 11 | 218 | Cunha et al., 2003, Coutinho et al., 2011b |
| | IST4110 | 9/25/01 | *B. cenocepacia* IIIA | - | A3 | RAPD02 | 11 | 614 | Cunha et al., 2003, Coutinho et al., 2011b |
| | IST4112 | 10/11/01 | *B. cenocepacia* IIIA | - | A3 | RAPD02 | 11 | 614 | Cunha et al., 2003, Coutinho et al., 2011b |
| | IST4113 | 11/6/01 | *B. cenocepacia* IIIA | - | A3 | RAPD02 | 11 | 614 | Cunha et al., 2003, Coutinho et al., 2011b |
| | IST4119 | 1/22/02 | *B. multivorans* | + | E1 | ND | 9 | ND | Cunha et al., 2003 |
| | IST4116A | 2/11/02 | *B. cenocepacia* IIIA | - | A3 | RAPD02 | 11 | 614 | Cunha et al., 2003, Coutinho et al., 2011b |
| | IST4116B | 2/11/02 | *B. cenocepacia* IIIA | - | A3 | RAPD02 | 11 | 218 | Cunha et al., 2003, Coutinho et al., 2011b |
| | IST4131 | 2/26/02 | *B. cenocepacia* IIIA | - | A3 | RAPD02 | 11 | 218 | Cunha et al., 2003, Coutinho et al., 2011b |
| | IST4129 | 3/26/02 | *B. cenocepacia* IIIA | - | A3 | RAPD02 | 11 | 218 | Cunha et al., 2003, Coutinho et al., 2011b |
| | IST4130 | 5/14/02 | *B. cenocepacia* IIIA | - | A3 | RAPD02 | 11 | 218 | Cunha et al., 2003, Coutinho et al., 2011b |
| | IST4134 | 7/2/02 | *B. cenocepacia* IIIA | - | A3 | RAPD02 | 11 | 218 | Cunha et al., 2003, Coutinho et al., 2011b |

| | | | | | | | | | |
|---|---|---|---|---|---|---|---|---|---|
| **Patient N** | IST431A | 8/30/98 | *B. cepacia* | + | C2 | ND | ND | ND | This work |
| | IST431B | 8/30/98 | *B. cepacia* | + | C2 | ND | ND | ND | This work |
| | IST443A | 3/3/99 | *B. cepacia* | + | C2 | ND | ND | ND | This work |
| | IST443B | 3/3/99 | *B. cepacia* | + | C2 | ND | ND | ND | This work |
| | IST443C | 3/3/99 | *B. cepacia* | + | C2 | ND | ND | ND | This work |
| | IST443D | 3/3/99 | *B. cepacia* | + | C2 | ND | ND | ND | This work |
| | IST444A | 3/3/99 | *B. cepacia* | + | C2 | ND | ND | ND | This work |
| | IST444B | 3/3/99 | *B. cepacia* | + | C2 | ND | ND | ND | This work |
| | IST444C | 3/3/99 | *B. cepacia* | + | C2 | ND | ND | ND | This work |
| | IST449A | 6/9/99 | *B. cepacia* | + | C2 | ND | ND | ND | This work |
| | IST449B | 6/9/99 | *B. cepacia* | + | C2 | ND | ND | ND | This work |
| | IST463A | 5/23/00 | *B. multivorans* | + | - | ND | ND | ND | This work |
| | IST463B | 5/23/00 | *B. multivorans* | + | - | ND | ND | ND | This work |
| | IST472A | 10/18/00 | *B. cepacia* | + | C2 | ND | ND | ND | This work |
| | IST472B | 10/18/00 | *B. cepacia* | + | C2 | ND | ND | ND | This work |
| | IST485A | 4/1/01 | *B. cepacia* | + | C2 | ND | ND | ND | This work |
| | IST485B | 4/1/01 | *B. cepacia* | + | C2 | ND | ND | ND | This work |
| | IST485C | 4/1/01 | *B. cepacia* | + | C2 | ND | ND | ND | This work |
| | IST491A | 4/26/01 | *B. cepacia* | + | C2 | ND | 12 | ND | This work |
| | IST4104A | 8/16/01 | *B. cepacia* | + | C2 | ND | ND | ND | This work |
| | IST4104B | 8/16/01 | *B. cepacia* | + | C2 | ND | ND | ND | This work |
| | IST4105A | 8/31/01 | *B. cepacia* | + | C2 | ND | ND | ND | This work |
| | IST4105B | 8/31/01 | *B. cepacia* | + | C2 | ND | ND | ND | This work |
| | IST4106A | 9/24/01 | *B. cepacia* | + | C2 | ND | ND | ND | This work |
| | IST4106B | 9/24/01 | *B. cepacia* | + | C2 | ND | ND | ND | This work |
| | IST4106C | 9/24/01 | *B. cepacia* | + | C2 | ND | ND | ND | This work |
| | IST4115A | 11/8/01 | *B. cepacia* | + | C2 | ND | ND | ND | This work |
| | IST4115B | 11/8/01 | *B. cepacia* | + | C2 | ND | ND | ND | This work |
| | IST4117A | 1/3/02 | *B. cepacia* | + | C2 | ND | ND | ND | This work |
| | IST4117B | 1/3/02 | *B. cepacia* | + | C2 | ND | ND | ND | This work |
| Patient O | IST430 | 9/30/98 | *B. cenocepacia* IIIA | + | A4 | RAPD03 | 11 | ND | Cunha et al., 2003 |
| | IST435 | 11/1/98 | *B. cenocepacia* IIIB | + | B1 | RAPD09 | 14 | ND | Cunha et al., 2003 |
| | IST440 | 2/8/99 | *B. cenocepacia* IIIB | + | B1 | RAPD09 | 14 | ND | Cunha et al., 2003 |
| | IST450 | 6/9/99 | *B. cenocepacia* IIIB | + | B1 | RAPD09 | 14 | ND | Cunha et al., 2003 |
| | IST458 | 3/8/00 | *B. cenocepacia* IIIB | + | B1 | RAPD09 | 14 | ND | Cunha et al., 2003 |
| | IST462 | 5/23/00 | *B. cenocepacia* IIIA | + | A4 | RAPD03 | 11 | 280 | Cunha et al., 2003 |
| | IST463 | 5/23/00 | *B. cenocepacia* IIIA | + | A4 | RAPD03 | 11 | ND | Cunha et al., 2003 |
| | IST467 | 7/19/00 | *B. cenocepacia* IIIA | + | A4 | RAPD03 | 11 | ND | Cunha et al., 2003 |
| | IST470 | 10/18/00 | *B. cenocepacia* IIIA | + | A4 | RAPD03 | 11 | ND | Cunha et al., 2003 |
| | IST471 | 10/18/00 | *B. cenocepacia* IIIB | + | B1 | RAPD09 | 14 | ND | Cunha et al., 2003 |
| | IST478 | 2/15/01 | *B. cenocepacia* IIIA | + | A4 | RAPD03 | 11 | ND | Cunha et al., 2003 |
| | IST486 | 4/3/01 | *B. cenocepacia* IIIA | + | A4 | RAPD03 | 11 | ND | Cunha et al., 2003 |
| | IST490 | 4/26/01 | *B. cenocepacia* IIIA | + | A4 | RAPD03 | 11 | ND | Cunha et al., 2003 |
| | IST497 | 5/31/01 | *B. cenocepacia* IIIA | + | A4 | RAPD03 | 11 | ND | Cunha et al., 2003 |
| | IST4107 | 9/11/01 | *B. cenocepacia* IIIA | + | A4 | RAPD03 | 11 | ND | Cunha et al., 2003 |
| | IST4114 | 11/8/01 | *B. cenocepacia* IIIB | + | B1 | RAPD09 | 14 | ND | Cunha et al., 2003 |
| | IST4118 | 1/3/02 | *B. cenocepacia* IIIB | + | B1 | RAPD09 | 14 | ND | Cunha et al., 2003 |
| | IST4123 | 2/28/02 | *B. cenocepacia* IIIB | + | B1 | RAPD09 | 14 | ND | Cunha et al., 2003 |
| | IST4128 | 5/2/02 | *B. cepacia* | + | C2 | ND | 12 | ND | Cunha et al., 2003 |
| | IST4135 | 11/21/02 | *B. cepacia* | + | C2 | ND | 12 | ND | Cunha et al., 2007 |
| | IST4137 | 1/23/03 | *B. cepacia* | + | C2 | ND | 12 | ND | Cunha et al., 2007 |
| | IST4139 | 1/24/03 | *B. cepacia* | + | C2 | ND | 12 | ND | Cunha et al., 2007 |
| | IST4142 | 1/25/03 | *B. cepacia* | + | C2 | ND | 12 | ND | Cunha et al., 2007 |
| | IST4146 | 8/7/03 | *B. cepacia* | + | C2 | ND | 12 | ND | Cunha et al., 2007 |
| | IST4150 | 9/30/03 | *B. cepacia* | + | C2 | ND | 12 | ND | Cunha et al., 2007 |

| | | | | | | | | | |
|---|---|---|---|---|---|---|---|---|---|
| | IST4157 | 1/22/04 | *B. cepacia* | + | C2 | ND | 12 | ND | Cunha et al., 2007 |
| | IST4165 | 3/25/04 | *B. cepacia* | + | C2 | ND | 12 | ND | Cunha et al., 2007 |
| | IST4167 | 5/13/04 | *B. cepacia* | + | C2 | ND | 12 | ND | Cunha et al., 2007 |
| | IST4240 | 9/15/04 | *B. cepacia* | + | C2 | ND | 12 | ND | Cunha et al., 2007 |
| | IST4177A | 11/17/04 | *B. cenocepacia* IIIB | + | B2 | RAPD10 | ND | ND | This work |
| | IST4180 | 1/26/05 | *B. cepacia* | + | C2 | ND | 12 | ND | Cunha et al., 2007 |
| | IST4183 | 3/16/05 | *B. cepacia* | + | C2 | ND | 12 | ND | Cunha et al., 2007 |
| | IST4189 | 5/25/05 | *B. cepacia* | + | C2 | ND | 12 | ND | Cunha et al., 2007 |
| | IST4204A | 7/27/05 | *B. cenocepacia* IIIB | + | B2 | RAPD10 | ND | ND | This work |
| | IST4209 | 9/21/05 | *B. cepacia* | + | C2 | ND | 12 | ND | Cunha et al., 2007 |
| | IST4216 | 11/16/05 | *B. cepacia* | + | C2 | ND | 12 | ND | Cunha et al., 2007 |
| | IST4227 | 1/18/06 | *B. cepacia* | + | C2 | ND | 12 | ND | Cunha et al., 2007 |
| | IST4230 | 2/8/06 | *B. cepacia* | + | C2 | ND | 12 | ND | Cunha et al., 2007 |
| | IST4297 | 3/21/07 | *B. cepacia* | + | C2 | ND | ND | ND | This work |
| | IST4327 | 4/10/07 | *B. cepacia* | + | C2 | ND | ND | ND | This work |
| | IST4315 | 4/20/07 | *B. cepacia* | + | C2 | ND | ND | ND | This work |
| | IST4355 | 7/4/07 | *B. cepacia* | + | C2 | ND | ND | ND | This work |
| | IST4369 | 7/12/07 | *B. cepacia* | + | C2 | ND | ND | ND | This work |
| | IST4383 | 10/31/07 | *B. cepacia* | + | C2 | ND | ND | ND | This work |
| | IST432 | 10/22/98 | *B. cenocepacia* IIIA | + | A5 | RAPD04 | 13 | ND | Cunha et al., 2003 |
| **Patient P** | IST468 | 10/18/00 | *B. cenocepacia* IIIA | + | A5 | RAPD04 | 13 | ND | Cunha et al., 2003 |
| | IST489 | 4/19/01 | *B. cenocepacia* IIIA | + | A5 | RAPD04 | 13 | ND | Cunha et al., 2003 |
| | IST438 | 1/25/99 | *B. cenocepacia* IIIB | + | B3 | RAPD10 | 15 | 43 | Cunha et al., 2003 |
| | IST445 | 3/8/99 | *B. cenocepacia* IIIB | + | B3 | RAPD10 | 15 | 43 | Cunha et al., 2003 |
| | IST452 | 7/12/99 | *B. cenocepacia* IIIB | + | B3 | RAPD10 | 15 | 43 | Cunha et al., 2003 |
| | IST454 | 12/22/99 | *B. cenocepacia* IIIB | + | B3 | RAPD10 | 15 | ND | Cunha et al., 2003 |
| | IST456 | 3/8/00 | *B. cenocepacia* IIIB | + | B3 | RAPD10 | 15 | 43 | Cunha et al., 2003 |
| | IST465 | 5/31/00 | *B. cenocepacia* IIIB | + | B3 | RAPD10 | 15 | ND | Cunha et al., 2003 |
| | IST474 | 1/9/01 | *B. cenocepacia* IIIB | + | B3 | RAPD10 | 15 | ND | Cunha et al., 2003 |
| | IST475 | 1/16/01 | *B. cenocepacia* IIIB | + | B3 | RAPD10 | 15 | 43 | Cunha et al., 2003 |
| | IST476 | 1/19/01 | *B. cenocepacia* IIIB | + | B3 | RAPD10 | 15 | ND | Cunha et al., 2003 |
| | IST484 | 3/29/01 | *B. cenocepacia* IIIB | + | B3 | RAPD10 | 15 | ND | Cunha et al., 2003 |
| | IST4144 | 7/10/03 | *B. cenocepacia* IIIB | + | B3 | RAPD10 | 15 | ND | Cunha et al., 2007 |
| | IST4149 | 9/25/03 | *B. cenocepacia* IIIB | + | B3 | RAPD10 | 15 | ND | Cunha et al., 2007 |
| **Patient R** | IST4155 | 11/27/03 | *B. cenocepacia* IIIB | - | B4 | RAPD10 | 15 | ND | Cunha et al., 2007 |
| | IST4164 | 3/11/04 | *B. cenocepacia* IIIB | - | B4 | RAPD10 | 15 | ND | Cunha et al., 2007 |
| | IST4178 | 11/17/04 | *B. cenocepacia* IIIB | + | B3 | RAPD10 | 15 | ND | Cunha et al., 2007 |
| | IST4203 | 7/28/05 | *B. cenocepacia* IIIB | + | B3 | RAPD10 | 15 | ND | Cunha et al., 2007 |
| | IST4205 | 8/10/05 | *B. cenocepacia* IIIB | + | B3 | RAPD10 | 15 | ND | Cunha et al., 2007 |
| | IST4210 | 9/21/05 | *B. cenocepacia* IIIB | + | B3 | RAPD10 | 15 | ND | Cunha et al., 2007 |
| | IST4219 | 11/16/05 | *B. cenocepacia* IIIB | + | B3 | RAPD10 | 15 | ND | Cunha et al., 2007 |
| | IST4228 | 1/18/06 | *B. cenocepacia* IIIB | + | B3 | RAPD10 | 15 | ND | Cunha et al., 2007 |
| | IST4231 | 2/14/06 | *B. cenocepacia* IIIB | + | B3 | RAPD10 | 15 | ND | Cunha et al., 2007 |
| | IST4232 | 2/21/06 | *B. cenocepacia* IIIB | + | B3 | RAPD10 | 15 | ND | Cunha et al., 2007 |
| | IST4235 | 3/2/06 | *B. cenocepacia* IIIB | + | B3 | RAPD10 | 15 | ND | Cunha et al., 2007 |
| | IST4252 | 6/14/06 | *B. cenocepacia* IIIB | + | B3 | RAPD10 | ND | ND | This work |
| | IST4255 | 7/12/06 | *B. cenocepacia* IIIB | + | B3 | RAPD10 | ND | ND | This work |
| | IST466 | 6/12/00 | *B. cenocepacia* IIIB | + | B5 | RAPD11 | 16 | ND | Cunha et al., 2003 |
| | IST469 | 10/18/00 | *B. cenocepacia* IIIB | + | B5 | RAPD11 | 16 | ND | Cunha et al., 2003 |
| | IST473 | 12/20/00 | *B. cenocepacia* IIIB | + | B5 | RAPD11 | 16 | ND | Cunha et al., 2003 |
| **Patient T** | IST479 | 2/19/01 | *B. cenocepacia* IIIB | + | B5 | RAPD11 | 16 | ND | Cunha et al., 2003 |
| | IST4102 | 7/12/01 | *B. cenocepacia* IIIB | + | B5 | RAPD11 | 16 | ND | Cunha et al., 2003 |
| | IST4109 | 9/26/01 | *B. cenocepacia* IIIB | + | B5 | RAPD11 | 16 | ND | Cunha et al., 2003 |
| | IST481 | 2/21/01 | *B. contaminans* | + | D1 | ND | 17 | ND | Cunha et al., 2003 |

| | | | | | | | | | |
|---|---|---|---|---|---|---|---|---|---|
| | IST4148 | 9/3/03 | *B. contaminans* | + | D1 | ND | 17 | ND | Cunha et al., 2007 |
| | IST4169 | 6/8/04 | *B. contaminans* | + | D1 | ND | 17 | ND | Cunha et al., 2007 |
| | IST4241 | 6/28/04 | *B. contaminans* | + | D1 | ND | 17 | ND | Cunha et al., 2007 |
| | IST4200 | 7/19/05 | *B. contaminans* | + | D1 | ND | 17 | ND | Cunha et al., 2007 |
| | IST4224 | 12/27/05 | *B. contaminans* | + | D1 | ND | 17 | ND | Cunha et al., 2007 |
| **Patient V** | IST4256 | 7/14/06 | *B. contaminans* | + | D1 | ND | ND | ND | This work |
| | IST4323 | 4/24/07 | *B. contaminans* | + | D1 | ND | ND | ND | This work |
| | IST4538 | 10/4/10 | *B. contaminans* | + | D1 | ND | ND | ND | This work |
| | IST4657 | 6/14/11 | *B. contaminans* | + | D1 | ND | ND | ND | This work |
| | IST4684 | 10/12/11 | *B. contaminans* | + | D1 | ND | ND | ND | This work |
| | IST4751 | 10/17/12 | *B. contaminans* | + | D1 | ND | ND | ND | This work |
| | IST4920 | 4/8/16 | *B. contaminans* | + | D1 | ND | ND | ND | This work |
| | IST4121 | 1/31/02 | *B. cenocepacia* IIIA | + | A6 | RAPD05 | 7 | ND | Cunha et al., 2007 |
| | IST4136 | 1/23/03 | *B. cenocepacia* IIIA | + | A6 | RAPD05 | 7 | ND | Cunha et al., 2007 |
| | IST4140 | 5/15/03 | *B. cenocepacia* IIIA | + | A6 | RAPD05 | 7 | ND | Cunha et al., 2007 |
| | IST4141 | 5/15/03 | *B. cenocepacia* IIIA | + | A6 | RAPD05 | 7 | ND | Cunha et al., 2007 |
| | IST4151 | 10/28/03 | *B. cenocepacia* IIIA | + | A6 | RAPD05 | 7 | ND | Cunha et al., 2007 |
| | IST4153 | 11/15/03 | *B. cenocepacia* IIIA | + | A6 | RAPD05 | 7 | ND | Cunha et al., 2007 |
| | IST4154 | 11/15/03 | *B. cenocepacia* IIIA | + | A6 | RAPD05 | 7 | ND | Cunha et al., 2007 |
| | IST4166 | 4/18/04 | *B. cenocepacia* IIIA | + | A6 | RAPD05 | 7 | ND | Cunha et al., 2007 |
| | IST4170 | 6/24/04 | *B. cenocepacia* IIIA | + | A6 | RAPD05 | 7 | ND | Cunha et al., 2007 |
| | IST4892B | 6/24/04 | *B. cenocepacia* IIIA | + | A6 | RAPD05 | ND | ND | Moreira et al., 2017 |
| | IST4173 | 8/5/04 | *B. cepacia* | + | - | - | 19 | ND | Cunha et al., 2007 |
| | IST4179 | 1/3/05 | *B. cenocepacia* IIIA | + | A6 | RAPD05 | 7 | ND | Cunha et al., 2007 |
| | IST4182 | 3/9/05 | *B. cenocepacia* IIIA | + | A6 | RAPD05 | 7 | ND | Cunha et al., 2007 |
| | IST4187 | 4/20/05 | *B. cenocepacia* IIIA | + | A6 | RAPD05 | 7 | ND | Cunha et al., 2007 |
| | IST4191 | 6/8/05 | *B. cenocepacia* IIIA | + | A6 | RAPD05 | 7 | ND | Cunha et al., 2007 |
| | IST4202 | 7/27/05 | *B. cenocepacia* IIIA | + | A6 | RAPD05 | 7 | ND | Cunha et al., 2007 |
| | IST4213 | 10/6/05 | *B. cenocepacia* IIIA | + | A6 | RAPD05 | 7 | ND | Cunha et al., 2007 |
| | IST4234 | 3/2/06 | *B. cenocepacia* IIIA | + | A6 | RAPD05 | 7 | ND | Cunha et al., 2007 |
| | IST4259 | 8/5/06 | *B. cenocepacia* IIIA | - | A7 | RAPD05 | ND | ND | This work |
| | IST4893 | 8/5/06 | *B. cenocepacia* IIIA | - | A7 | RAPD05 | ND | ND | Moreira et al., 2017 |
| | IST4894 | 8/5/06 | *B. cenocepacia* IIIA | - | A7 | RAPD05 | ND | ND | Moreira et al., 2017 |
| | IST4346 | 6/28/07 | *B. cenocepacia* IIIA | - | A7 | RAPD05 | ND | ND | This work |
| | IST4417 | 10/9/08 | *B. cenocepacia* IIIA | - | A7 | RAPD05 | ND | ND | Moreira et al., 2017 |
| Patient AB | IST4895 | 10/9/08 | *B. cenocepacia* IIIA | - | A7 | RAPD05 | ND | ND | Moreira et al., 2017 |
| | IST4436 | 12/10/09 | *B. cenocepacia* IIIA | - | A7 | RAPD05 | ND | ND | This work |
| | IST4450 | 3/4/10 | *B. cenocepacia* IIIA | - | A7 | RAPD05 | ND | ND | This work |
| | IST4470 | 5/6/10 | *B. cenocepacia* IIIA | - | A7 | RAPD05 | ND | ND | This work |
| | IST4559 | 7/22/10 | *B. cenocepacia* IIIA | - | A7 | RAPD05 | ND | ND | This work |
| | IST4589 | 12/9/10 | *B. cenocepacia* IIIA | - | A7 | RAPD05 | ND | ND | This work |
| | IST4624 | 2/17/11 | *B. cenocepacia* IIIA | - | A7 | RAPD05 | ND | ND | This work |
| | IST4653 | 6/14/11 | *B. cenocepacia* IIIA | - | A7 | RAPD05 | ND | ND | This work |
| | IST4676 | 10/13/11 | *B. cenocepacia* IIIA | - | A7 | RAPD05 | ND | ND | This work |
| | IST4676S | 10/13/11 | *B. cenocepacia* IIIA | - | A7 | RAPD05 | ND | ND | Moreira et al., 2017 |
| | IST4676R | 10/13/11 | *B. cenocepacia* IIIA | - | A7 | RAPD05 | ND | ND | Moreira et al., 2017 |
| | IST4677S | 10/13/11 | *B. cenocepacia* IIIA | - | A7 | RAPD05 | ND | ND | Moreira et al., 2017 |
| | IST4725 | 3/29/12 | *B. cenocepacia* IIIA | - | A7 | RAPD05 | ND | ND | This work |
| | IST4787 | 2/7/13 | *B. cenocepacia* IIIA | - | A7 | RAPD05 | ND | ND | This work |
| | IST4797 | 4/26/13 | *B. cenocepacia* IIIA | - | A7 | RAPD05 | ND | ND | This work |
| | IST4835 | 12/5/13 | *B. cenocepacia* IIIA | - | A7 | RAPD05 | ND | ND | This work |
| | IST4854 | 4/24/14 | *B. cenocepacia* IIIA | - | A7 | RAPD05 | ND | ND | This work |
| | IST4882 | 8/21/14 | *B. cenocepacia* IIIA | - | A7 | RAPD05 | ND | ND | This work |
| | IST4882A | 8/21/14 | *B. cenocepacia* IIIA | - | A7 | RAPD05 | ND | ND | Moreira et al., 2017 |
| | IST4882B | 8/21/14 | *B. cenocepacia* IIIA | - | A7 | RAPD05 | ND | ND | Moreira et al., 2017 |

| | | | | | | | | | |
|---|---|---|---|---|---|---|---|---|---|
| | IST4884A | 8/21/14 | *B. cenocepacia* IIIA | - | A7 | RAPD05 | ND | ND | Moreira et al., 2017 |
| | IST4889 | 6/16/15 | *B. cenocepacia IIIA* | - | A7 | RAPD05 | ND | ND | This work |
| | IST4930 | 7/12/16 | *B. cenocepacia IIIA* | - | A7 | RAPD05 | ND | ND | This work |
| | IST4934 | 9/20/16 | *B. cenocepacia IIIA* | - | A7 | RAPD05 | ND | ND | This work |
| | IST4193 | 12/9/04 | *B. contaminans* | + | D2 | ND | 2 | 96 | Coutinho et al., 2015 |
| | IST4194 | 2/10/05 | *B. contaminans* | + | D2 | ND | ND | ND | Coutinho et al., 2015 |
| | IST4186 | 4/14/05 | *B. contaminans* | + | D2 | ND | ND | ND | Coutinho et al., 2015 |
| | IST4188 | 5/4/05 | *B. contaminans* | + | D2 | ND | ND | ND | Coutinho et al., 2015 |
| | IST4192 | 6/30/05 | *B. contaminans* | + | D2 | ND | ND | ND | Coutinho et al., 2015 |
| | IST4206 | 8/10/05 | *B. contaminans* | + | D2 | ND | ND | ND | Coutinho et al., 2015 |
| | IST4207 | 8/10/05 | *B. cenocepacia IIIB* | + | - | ND | ND | ND | Coutinho et al., 2015 |
| | IST4221 | 11/23/05 | *B. cepacia* | + | - | ND | ND | ND | Coutinho et al., 2015 |
| | IST4238 | 3/14/06 | *B. contaminans* | + | D2 | ND | ND | ND | Coutinho et al., 2015 |
| | IST4237 | 3/20/06 | *B. contaminans* | + | D2 | ND | ND | ND | Coutinho et al., 2015 |
| | IST4240a | 5/10/06 | *B. cenocepacia IIIA* | + | A8 | RAPD06 | ND | ND | Coutinho et al., 2015 |
| | IST4240b | 5/10/06 | *B. contaminans* | + | D2 | ND | ND | ND | Coutinho et al., 2015 |
| | IST4240c | 5/10/06 | *B. cepacia* | + | - | ND | ND | ND | Coutinho et al., 2015 |
| | IST4251 | 6/14/06 | *B. cenocepacia IIIA* | + | A8 | RAPD06 | ND | ND | Coutinho et al., 2015 |
| | IST4253a | 7/7/06 | *B. cenocepacia IIIA* | - | A10 | RAPD06 | ND | ND | Coutinho et al., 2015 |
| | IST4253b | 7/7/06 | *B. contaminans* | + | D2 | ND | ND | ND | Coutinho et al., 2015 |
| | IST4258a | 8/2/06 | *B. cenocepacia IIIA* | - | A10 | RAPD06 | ND | ND | Coutinho et al., 2015 |
| | IST4258b | 8/2/06 | *B. contaminans* | + | D2 | ND | ND | ND | Coutinho et al., 2015 |
| | IST4272 | 12/11/06 | *B. cenocepacia IIIA* | + | A9 | RAPD07 | ND | ND | Coutinho et al., 2015 |
| | IST4276 | 11/22/06 | *B. contaminans* | + | D2 | ND | ND | ND | Coutinho et al., 2015 |
| **Patient AF** | IST4303 | 3/28/07 | *B. contaminans* | + | D2 | ND | ND | ND | Coutinho et al., 2015 |
| | IST4319 | 4/23/07 | *B. contaminans* | + | D2 | ND | ND | ND | Coutinho et al., 2015 |
| | IST4322 | 4/23/07 | *B. contaminans* | + | D2 | ND | ND | ND | Coutinho et al., 2015 |
| | IST4391 | 3/19/08 | *B. contaminans* | + | D2 | ND | ND | ND | Coutinho et al., 2015 |
| | IST4402a | 7/9/08 | *B. cenocepacia IIIA* | - | A10 | RAPD06 | ND | ND | Coutinho et al., 2015 |
| | IST4402b | 7/9/08 | *B. contaminans* | + | D2 | ND | ND | ND | Coutinho et al., 2015 |
| | IST4408 | 8/13/08 | *B. cenocepacia IIIA* | - | A10 | RAPD06 | ND | ND | Coutinho et al., 2015 |
| | IST4412 | 9/16/08 | *B. contaminans* | + | D2 | ND | ND | ND | Coutinho et al., 2015 |
| | IST4420a | 10/17/08 | *B. cenocepacia IIIA* | - | A10 | RAPD06 | ND | ND | Coutinho et al., 2015 |
| | IST4420d | 10/17/08 | *B. dolosa* | + | - | ND | ND | ND | Coutinho et al., 2015 |
| | IST4435 | 11/23/09 | *B. contaminans* | + | D2 | ND | ND | ND | Coutinho et al., 2015 |
| | IST4497 | 1/11/10 | *B. contaminans* | + | D2 | ND | ND | ND | Coutinho et al., 2015 |
| | IST4456 | 3/29/10 | *B. contaminans* | + | D2 | ND | ND | ND | Coutinho et al., 2015 |
| | IST4474 | 5/18/10 | *B. contaminans* | + | D2 | ND | ND | ND | Coutinho et al., 2015 |
| | IST4496 | 2/22/10 | *B. contaminans* | + | D2 | ND | ND | ND | Coutinho et al., 2015 |
| | IST4485 | 5/10/10 | *B. contaminans* | + | D2 | ND | ND | ND | Coutinho et al., 2015 |
| | IST4490 | 6/16/10 | *B. contaminans* | + | D2 | ND | ND | ND | Coutinho et al., 2015 |
| | IST4550 | 7/19/10 | *B. contaminans* | + | D2 | ND | ND | ND | Coutinho et al., 2015 |
| | IST4513 | 8/9/10 | *B. contaminans* | + | D2 | ND | ND | ND | Coutinho et al., 2015 |
| | IST4517 | 10/10/10 | *B. contaminans* | + | D2 | ND | ND | ND | Coutinho et al., 2015 |
| | IST4566 | 10/29/10 | *B. contaminans* | + | D2 | ND | ND | ND | Coutinho et al., 2015 |
| | IST4152 | 10/30/03 | *B. cepacia* | + | C1 | RAPD12 | 12 | ND | Cunha et al., 2007 |
| | IST4158 | 2/12/04 | *B. cepacia* | + | C1 | RAPD12 | 12 | ND | Cunha et al., 2007 |
| | IST4159 | 3/2/04 | *B. cepacia* | + | C1 | RAPD12 | 12 | ND | Cunha et al., 2007 |
| | IST4160 | 4/2/04 | *B. cepacia* | + | C1 | RAPD12 | 12 | ND | Cunha et al., 2007 |
| | IST4168 | 5/18/04 | *B. cepacia* | + | C1 | RAPD12 | 12 | ND | Cunha et al., 2007 |
| **Patient AL** | IST4175 | 10/6/04 | *B. cepacia* | + | C1 | RAPD12 | 12 | ND | Cunha et al., 2007 |
| | IST4184 | 3/17/05 | *B. cepacia* | + | C1 | RAPD12 | 12 | ND | Cunha et al., 2007 |
| | IST4220 | 11/16/05 | *B. cepacia* | + | C1 | RAPD12 | 12 | ND | Cunha et al., 2007 |
| | IST4222 | 12/14/05 | *B. cepacia* | + | C1 | RAPD12 | 12 | ND | Cunha et al., 2007 |
| | IST4226 | 1/18/06 | *B. cepacia* | + | C1 | RAPD12 | 12 | ND | Cunha et al., 2007 |

| | | | | | | | | | |
|---|---|---|---|---|---|---|---|---|---|
| | IST4197 | 5/25/05 | *B. cenocepacia* IIIA | + | A11 | RAPD08 | 21 | **ND** | Cunha et al., 2007 |
| | IST4896 | 5/25/05 | *B. cenocepacia* IIIA | + | A11 | RAPD08 | ND | ND | Moreira et al., 2017 |
| | IST4190 | 6/1/05 | *B. cenocepacia* IIIA | + | A11 | RAPD08 | 21 | **ND** | Cunha et al., 2007 |
| | IST4201 | 7/21/05 | *B. cenocepacia* IIIA | + | A11 | RAPD08 | 21 | **ND** | Cunha et al., 2007 |
| | IST4211 | 9/22/05 | *B. cenocepacia* IIIA | + | A11 | RAPD08 | 21 | **ND** | Cunha et al., 2007 |
| | IST4215A | 10/27/05 | *B. cenocepacia IIIB* | + | - | ND | ND | ND | This work |
| | IST4223A | 12/15/05 | *B. cenocepacia* IIIA | + | A11 | RAPD08 | ND | ND | This work |
| | IST4223B | 12/15/05 | *B. multivorans* | + | - | ND | ND | ND | This work |
| | IST4243 | 5/11/06 | *B. cenocepacia* IIIA | + | A11 | RAPD08 | ND | ND | Moreira et al., 2017 |
| | IST4897 | 5/11/06 | *B. cenocepacia* IIIA | + | A11 | RAPD08 | ND | ND | Moreira et al., 2017 |
| | IST4304A | 3/29/07 | *B. cenocepacia* IIIA | + | A11 | RAPD08 | ND | ND | This work |
| | IST4304B | 3/29/07 | *B. multivorans* | + | - | ND | ND | ND | This work |
| | IST4364 | 6/21/07 | *B. cenocepacia* IIIA | + | A11 | RAPD08 | ND | ND | This work |
| | IST4386 | 11/22/07 | *B. cenocepacia* IIIA | - | A12 | RAPD08 | ND | ND | Moreira et al., 2017 |
| | IST4388 | 1/10/08 | *B. cenocepacia* IIIA | - | A12 | RAPD08 | ND | ND | This work |
| | IST4410 | 8/21/08 | *B. cenocepacia* IIIA | - | A12 | RAPD08 | ND | ND | Moreira et al., 2017 |
| | IST4898B | 8/21/08 | *B. cenocepacia* IIIA | - | A12 | RAPD08 | ND | ND | Moreira et al., 2017 |
| **Patient AN** | IST4442 | 10/8/09 | *B. cenocepacia* IIIA | - | A12 | RAPD08 | ND | ND | This work |
| | IST4446 | 1/21/10 | *B. cenocepacia* IIIA | - | A12 | RAPD08 | ND | ND | This work |
| | IST4466 | 5/6/10 | *B. cenocepacia* IIIA | - | A12 | RAPD08 | ND | ND | This work |
| | IST4466S | 5/6/10 | *B. cenocepacia* IIIA | - | A12 | RAPD08 | ND | ND | Moreira et al., 2017 |
| | IST4466R | 5/6/10 | *B. cenocepacia* IIIA | - | A12 | RAPD08 | ND | ND | Moreira et al., 2017 |
| | IST4469S | 5/6/10 | *B. cenocepacia* IIIA | - | A12 | RAPD08 | ND | ND | Moreira et al., 2017 |
| | IST4531 | 8/13/10 | *B. cenocepacia* IIIA | - | A12 | RAPD08 | ND | ND | This work |
| | IST4586 | 12/9/10 | *B. cenocepacia* IIIA | - | A12 | RAPD08 | ND | ND | This work |
| | IST4628 | 3/14/11 | *B. cenocepacia* IIIA | - | A12 | RAPD08 | ND | ND | This work |
| | IST4632 | 4/28/11 | *B. cenocepacia* IIIA | - | A12 | RAPD08 | ND | ND | This work |
| | IST4680 | 10/13/11 | *B. cenocepacia* IIIA | - | A12 | RAPD08 | ND | ND | This work |
| | IST4691 | 11/3/11 | *B. cenocepacia* IIIA | - | A12 | RAPD08 | ND | ND | This work |
| | IST4721A | 3/29/12 | *B. cenocepacia* IIIA | - | A12 | RAPD08 | ND | ND | This work |
| | IST4721B | 3/29/12 | *B. cenocepacia* IIIA | - | A12 | RAPD08 | ND | ND | This work |
| | IST4721 | 3/29/12 | *B. cenocepacia* IIIA | - | A12 | RAPD08 | ND | ND | Moreira et al., 2017 |
| | IST4722 | 3/29/12 | *B. cenocepacia* IIIA | - | A12 | RAPD08 | ND | ND | Moreira et al., 2017 |
| | IST4788 | 2/7/13 | *B. cenocepacia* IIIA | - | A12 | RAPD08 | ND | ND | Moreira et al., 2017 |
| | IST4199 | 7/14/05 | *B. cepacia* | + | C1 | RAPD12 | 12 | ND | Cunha et al., 2007 |
| | IST4218 | 11/17/05 | *B. cepacia* | + | C1 | RAPD12 | 12 | ND | Cunha et al., 2007 |
| **Patient AP** | IST4236 | 3/8/06 | *B. cepacia* | + | C1 | RAPD12 | 12 | ND | Cunha et al., 2007 |
| | IST4183 | 5/3/06 | *B. cepacia* | + | C1 | RAPD12 | ND | ND | This work |
| | IST4406 | 5/6/08 | *B. cepacia* | + | C1 | RAPD12 | ND | ND | This work |
| | IST4198 | 7/7/05 | *B. cepacia* | + | C5 | ND | 24 | ND | Cunha et al., 2007 |
| | IST4214 | 10/13/05 | *B. cepacia* | + | C5 | ND | 24 | ND | Cunha et al., 2007 |
| | IST4225 | 12/26/05 | *B. cepacia* | + | C5 | ND | 24 | ND | Cunha et al., 2007 |
| | IST4229 | 1/31/06 | *B. cepacia* | + | C5 | ND | 24 | ND | Cunha et al., 2007 |
| | IST4233 | 2/22/06 | *B. cepacia* | + | C5 | ND | 24 | ND | Cunha et al., 2007 |
| **Patient AQ** | IST4254 | 7/12/06 | *B. cepacia* | + | C5 | ND | ND | ND | This work |
| | IST4294 | 1/29/07 | *B. cepacia* | + | C5 | ND | ND | ND | This work |
| | IST4308 | 4/4/07 | *B. cepacia* | + | C5 | ND | ND | ND | This work |
| | IST4399 | 1/8/08 | *B. cepacia* | + | C5 | ND | ND | ND | This work |
| | IST4395 | 4/16/08 | *B. cenocpeacia IIIB* | - | - | ND | ND | ND | This work |
| | IST4407 | 6/18/08 | *B. cepacia* | + | C5 | ND | ND | ND | This work |
| | IST4208 | 8/25/05 | *B. dolosa* | - | F | ND | ND | 668 | Moreira et al., 2014 |
| | IST4370 | 7/12/07 | *B. dolosa* | - | F | ND | ND | 668 | Moreira et al., 2014 |
| | IST4377 | 8/13/07 | *B. dolosa* | - | F | ND | ND | 668 | Moreira et al., 2014 |
| | IST4385 | 11/22/07 | *B. dolosa* | - | F | ND | ND | 668 | Moreira et al., 2014 |

| | | | | | | | | | |
|---|---|---|---|---|---|---|---|---|---|
| **Patient AR** | IST4387 | 1/10/08 | *B. dolosa* | - | F | ND | ND | 668 | Moreira et al., 2014 |
| | IST4389 | 3/13/08 | *B. dolosa* | - | F | ND | ND | 668 | Moreira et al., 2014 |
| | IST4409 | 8/4/08 | *B. dolosa* | - | F | ND | ND | 668 | Moreira et al., 2014 |
| | IST4616 | 8/5/09 | *B. dolosa* | - | F | ND | ND | 668 | Moreira et al., 2014 |
| | IST4481 IIIB | 5/19/10 | *B. cenocepacia* IIIB | + | B6 | RAPD10 | ND | 43 | Moreira et al., 2014 |
| | IST4481 VI | 5/19/10 | *B. dolosa* | - | F | ND | ND | 668 | Moreira et al., 2014 |
| | IST4484 IIIB | 4/30/10 | *B. cenocepacia* IIIB | + | B6 | RAPD10 | ND | 43 | Moreira et al., 2014 |
| | IST4484 VI | 4/30/10 | *B. dolosa* | - | F | ND | ND | 668 | Moreira et al., 2014 |
| | IST4499 | 7/27/10 | *B. cenocepacia* IIIB | + | B6 | RAPD10 | ND | 43 | Moreira et al., 2014 |
| | IST4503 | 7/22/10 | *B. cenocepacia* IIIB | + | B6 | RAPD10 | ND | 43 | Moreira et al., 2014 |
| | IST4507 | 10/7/10 | *B. dolosa* | - | F | ND | ND | 668 | Moreira et al., 2014 |
| | IST4583 | 12/9/10 | *B. dolosa* | - | F | ND | ND | 668 | Moreira et al., 2014 |
| | IST4601S | 1/12/10 | *B. dolosa* | - | F | ND | ND | 668 | Moreira et al., 2014 |
| | IST4601R | 1/12/10 | *B. dolosa* | - | F | ND | ND | 668 | Moreira et al., 2014 |
| **Patient AU** | IST4283 | 2/7/07 | *B. cepacia* | + | C3 | RAPD12 | ND | ND | This work |
| | IST4299 | 3/21/07 | *B. cepacia* | + | C3 | RAPD12 | ND | ND | This work |
| | IST4371 | 7/11/07 | *B. cepacia* | + | C3 | RAPD12 | ND | ND | This work |
| | IST4378 | 9/12/07 | *B. cepacia* | + | C3 | RAPD12 | ND | ND | This work |
| | IST4384 | 11/7/07 | *B. cepacia* | + | C3 | RAPD12 | ND | ND | This work |
| | IST4392 | 4/8/08 | *B. cepacia* | + | C3 | RAPD12 | ND | ND | This work |
| | IST4401 | 6/18/08 | *B. cepacia* | + | C3 | RAPD12 | ND | ND | This work |
| | IST4413 | 9/17/08 | *B. cepacia* | + | C3 | RAPD12 | ND | ND | This work |
| | IST4422 | 10/15/08 | *B. cepacia* | + | C3 | RAPD12 | ND | ND | This work |
| | IST4424 | 11/12/08 | *B. cepacia* | + | C3 | RAPD12 | ND | ND | This work |
| | IST4433 | 7/6/09 | *B. cepacia* | + | C3 | RAPD12 | ND | ND | This work |
| | IST4527 | 10/18/10 | *B. cepacia* | + | C3 | RAPD12 | ND | ND | This work |
| | IST4541 | 6/14/10 | *B. cepacia* | + | C3 | RAPD12 | ND | ND | This work |
| | IST4546 | 7/19/10 | *B. cepacia* | + | C4 | RAPD13 | ND | ND | This work |
| | IST4597 | 12/20/10 | *B. cepacia* | + | C4 | RAPD13 | ND | ND | This work |
| | IST4604 | 1/24/10 | *B. cepacia* | + | C3 | RAPD12 | ND | ND | This work |
| | IST4623 | 2/7/11 | *B. cepacia* | + | C3 | RAPD12 | ND | ND | This work |
| | IST4640 | 5/2/11 | *B. cepacia* | + | C3 | RAPD12 | ND | ND | This work |
| | IST4665 | 6/27/11 | *B. cepacia* | + | C3 | RAPD12 | ND | ND | This work |
| | IST4673 | 10/10/11 | *B. cepacia* | + | C3 | RAPD12 | ND | ND | This work |
| | IST4709 | 12/9/11 | *B. cepacia* | + | C3 | RAPD12 | ND | ND | This work |
| | IST4729 | 4/16/12 | *B. cepacia* | + | C3 | RAPD12 | ND | ND | This work |
| | IST4746 | 9/10/12 | *B. cepacia* | + | C3 | RAPD12 | ND | ND | This work |
| | IST4755 | 10/29/12 | *B. cepacia* | + | C3 | RAPD12 | ND | ND | This work |
| | IST4768 | 11/26/12 | *B. cepacia* | + | C3 | RAPD12 | ND | ND | This work |
| | IST4776 | 1/21/13 | *B. cepacia* | + | C3 | RAPD12 | ND | ND | This work |
| | IST4793 | 3/18/13 | *B. cepacia* | + | C3 | RAPD12 | ND | ND | This work |
| | IST4815 | 6/24/13 | *B. cepacia* | + | C3 | RAPD12 | ND | ND | This work |
| | IST4832 | 11/18/13 | *B. cepacia* | + | C3 | RAPD12 | ND | ND | This work |
| | IST4844 | 1/13/14 | *B. cepacia* | + | C3 | RAPD12 | ND | ND | This work |
| | IST4919 | 4/2/16 | *B. cepacia* | + | C3 | RAPD12 | ND | ND | This work |
| | IST4923 | 5/9/16 | *B. cepacia* | + | C3 | RAPD12 | ND | ND | This work |
| | IST4928 | 6/6/16 | *B. cepacia* | + | C3 | RAPD12 | ND | ND | This work |
| | IST4933 | 9/12/16 | *B. cepacia* | + | C3 | RAPD12 | ND | ND | This work |
